# Prescription and Nonprescription Drug Use Among People With Eating Disorders

**DOI:** 10.1001/jamanetworkopen.2025.22406

**Published:** 2025-07-22

**Authors:** Sarah-Catherine Rodan, Sarah Maguire, Noah Meez, Kayla Greenstien, Garen Zartarian, Katherine L. Mills, Anastasia Suraev, Miguel A. Bedoya-Pérez, Iain S. McGregor

**Affiliations:** 1 Lambert Initiative for Cannabinoid Therapeutics, Brain and Mind Centre, The University of Sydney, Sydney, New South Wales, Australia; 2 School of Psychology, Faculty of Science, The University of Sydney, Sydney, New South Wales, Australia; 3 InsideOut Institute for Eating Disorders, Central Clinical School, Faculty of Medicine and Health, The University of Sydney, Sydney, New South Wales, Australia; 4 Sydney Local Health District, New South Wales Health, Sydney, New South Wales, Australia; 5 Faculty of Medicine and Health, The University of Sydney, Sydney, New South Wales, Australia; 6 University College London, London, United Kingdom; 7 Matilda Centre for Research in Mental Health and Substance Use, The University of Sydney, Sydney, New South Wales, Australia

## Abstract

**Importance:**

There are few effective pharmacotherapies for treating eating disorders (EDs). High rates of substance use among individuals with EDs suggest potential self-medication of symptoms.

**Objective:**

To explore the experiences of individuals with EDs regarding use of prescribed and nonprescribed drugs.

**Design, Setting, and Participants:**

This survey study assessed responses to the Medications and Other Drugs for Eating Disorders (MED-FED) survey, which was advertised internationally using social media, online forums, and clinical services and recruited adults who self-reported an ED or disordered eating from November 10, 2022, to May 31, 2023. The online survey queried about recent prescribed and nonprescribed drug use as well as the perceived benefits and harms of each substance.

**Exposures:**

EDs or disordered eating and co-occurring mental health conditions. Substances evaluated included caffeine, alcohol, nicotine, cannabis, prescription psychotropics, psychedelics, ketamine, 3,4-methylenedioxymethamphetamine (or ecstasy), stimulants, opioids, and other drugs.

**Main Outcomes and Measures:**

Respondents described drug use over the past 12 months. For each drug used, they rated their agreement or disagreement on a 5-point Likert scale (−2, strongly disagree; −1, disagree; 0, neutral; 1, agree; or 2, strongly agree) with the following 3 statements: (1) this medication/drug makes my eating disorder symptoms better; (2) this medication/drug has overall benefits for my mental health; and (3) this medication/drug has unpleasant side effects.

**Results:**

There were 7648 participants recruited, of whom 6612 completed the demographic portion, and 5123 completed the entire survey. Among the 6612 respondents (mean [SD] age, 24.3 [7.7] years), the sample was predominantly female (6217 [94.0%]), and most resided in Australia (1981 [30.0%]), the UK (1409 [21.3%]), or the US (1195 [18.0%]). Diagnosed EDs included 2696 (40.8%) individuals with anorexia nervosa, 1258 (19.0%) with bulimia nervosa, 757 (11.4%) with binge-eating disorder, and 589 (8.9%) with avoidant/restrictive food intake disorder. Many respondents (2493 [37.7%]) were undiagnosed. Psychiatric comorbidities were highly prevalent; depression was reported by 4333 respondents (65.5%). Cannabis and psychedelics were highest-rated for improving ED symptoms. Prescription antidepressants were rated highly for overall mental health but not for ED symptoms, with the exception of fluoxetine for bulimia nervosa and lisdexamfetamine for binge-eating disorder. Alcohol, nicotine, and tobacco were rated as the most harmful drugs.

**Conclusions and Relevance:**

The findings of this survey study of prescription and nonprescription drug use suggest that cannabis and psychedelics were perceived by survey respondents as efficacious in alleviating their ED symptoms, which supports further research in this area. Prescription psychotropics were perceived as being relatively ineffective for ED symptoms but beneficial to general mental health.

## Key Points

**Question:**

Which prescription and nonprescription drugs are used by individuals with eating disorders (EDs), and how are they associated with ED symptoms?

**Findings:**

In this survey study that recruited 7648 respondents self-reporting various EDs, cannabis and psychedelics were among a small set of drugs rated positively for relief of ED symptoms. Prescribed psychotropics were rated positively for overall mental health, while alcohol, nicotine, and tobacco were rated as having the greatest adverse effects.

**Meaning:**

The findings of this study suggest that few drugs have self-perceived benefits of treatment among individuals with EDs, while the therapeutic benefits of cannabis and psychedelics deserve further investigation.

## Teaser

This survey study explores the use of prescription and nonprescription drugs among respondents self-reporting their various eating disorders.

## Introduction

Eating disorders (EDs) are serious mental health conditions characterized by persistent disturbances in eating behaviors, related thoughts, and emotions.^1,2^ The *Diagnostic and Statistical Manual of Mental Disorders* (Fifth Edition) (*DSM-5*) currently identifies 8 relevant disorders with specific characteristics and symptoms: binge-eating disorder (BED), bulimia nervosa (BN), anorexia nervosa (AN), avoidant/restrictive food intake disorder (ARFID), other specified feeding or ED (OSFED), unspecified feeding or ED, rumination disorder, and pica.^3^ Disordered eating often develops during adolescence and is associated with physical, developmental, and psychosocial problems that substantially diminish quality of life.^4^ Death by suicide and owing to physical health complications is common,^5,6,7^ and economic costs can be profound.^8^

EDs are difficult conditions to manage and treat, with the majority of cases progressing into severe and enduring illness (>7 years’ duration).^9,10,11^ Psychological interventions dominate current treatment approaches since few prescription psychotropic drugs have demonstrable efficacy.^2^ Currently, only 2 medications have regulatory approval for EDs: lisdexamfetamine, a stimulant, for treating BED, and fluoxetine, an antidepressant, for treating BN.^1,2^ No pharmacologic treatments are approved for AN.^2^ Nevertheless, in clinical practice, patients with EDs are often prescribed psychotropic drugs to manage comorbid depression and anxiety.^2^

The co-occurrence of EDs with substance use and problematic use is well-documented in ED cohorts, particularly among individuals with binge–purge-type EDs.^12,13,14,15,16,17^ This may reflect self-medication for negative affective states or to obtain desired appetite-modulating effects of certain substances such as caffeine and stimulants.^13^ There is increasing interest in the therapeutic potential of cannabis and psychedelic drugs for individuals with EDs and preliminary supportive evidence.^18,19,20,21,22,23,24^

Overall, there is sparse information about the lived experience of people with EDs and drug use. To help address this, a comprehensive survey was designed that probed the use of prescription and nonprescription drug use in people with EDs. Respondents rated the perceived effects of these drugs on their ED symptoms and overall mental health as well as their adverse effects.

## Methods

This survey study assessed responses to the Medications and Other Drugs for Eating Disorders (MED-FED) survey, which was open from November 10, 2022, to May 31, 2023, and was advertised internationally using social media, online forums, and clinical services. Eligibility criteria included being 18 years or older, being confident in English, and self-reporting a current clinically diagnosed ED or disordered eating causing distress. Data were collected anonymously in REDCap.^25^ The survey took 15 to 30 minutes to complete. Participants provided informed consent online. The University of Sydney Human Research Ethics Committee provided ethical approval. The reporting of results adhered to guidelines outlined in the National Statement on Ethical Conduct in Human Research.^26^ The study followed the American Association for Public Opinion Research (AAPOR) reporting guideline.

Individual sociodemographic characteristics were self-reported using the survey instrument that included categories for race and ethnicity. Race and ethnicity data were collected to assess sample representativeness and potential differences in ED presentation and treatment across these variables. Race and ethnicity categories included Aboriginal, Asian, Black or African American, Hispanic or Latino, American Indian or Alaska Native, Pacific Islander, Torres Strait Islander, White, and other (chosen by respondents and described as Black other, Indian, Inuit, Jewish, Métis, Middle Eastern, Romani, White/Aboriginal, White/Asian, White/Black, White/Jewish, White/Latin American, White/Middle Eastern, White/Pacific Islander, White/Romani, multiracial, or other) or prefer not to say.

### Study Design and Measures

A multidisciplinary team of experts developed the survey. Initial sections queried about demographics, ED diagnosis, comorbidities, and history of treatments. Respondents who were undiagnosed nominated disordered eating symptoms recognized by the *DSM-5*. Statistical analysis of the perceived effects was limited to diagnostic categories with more than 150 respondents.

Survey sections queried about recent (the past 12 months) use of prescription medications, caffeine, alcohol, tobacco and nicotine, cannabis, stimulants, psychedelics, prosocial or party drugs (eg, 3,4-methylenedioxymethamphetamine [MDMA] or ecstasy, γ-hydroxybutyric acid [GHB]), opioids, and any other drugs. For each drug used, respondents reacted to the following 3 statements: (1) This medication/drug makes my eating disorder symptoms better. (2) This medication/drug has overall benefits for my mental health. (3) This medication/drug has unpleasant side effects. Responses were measured on a 5-point Likert scale (−2, strongly disagree; −1, disagree; 0, neutral; 1, agree; and 2, strongly agree).

For each drug used, respondents were asked, “Do you believe you have a problem with X?” and to nominate their 3 drugs of choice for (1) treating their ED and (2) overall, irrespective of ED effects. The full survey is presented in the eAppendix in Supplement 1.

### Statistical Analysis

χ^2^ Tests of independence compared the demographic and clinical categoric variables for each diagnostic group with all others combined. Fisher exact tests assessed the accuracy of *P* values when cell sizes were small (expected frequencies <5). The standardized residuals of the χ^2^ tests (*z* score) assessed which cells may have contributed to significant results. Standardized residuals that were more than 1.96 were deemed significant contributors. For age, a continuous variable, multiple Mann-Whitney tests were used to compare diagnostic groups with all others combined.

Binary logistic regression models were used to assess whether the ED type was associated with daily or past 12-month use of a substance. Ordinal logistic regression models investigated whether having a particular diagnosis when using a particular drug was associated with a higher or lower score on the Likert scale efficacy and tolerability metrics. When ordinal logistic regression detected a significant effect by diagnostic group, Mann-Whitney tests were used on the translated Likert scales (range, −2 to 2) to assess whether the distribution of responses differed for specific diagnostic groups relative to all other respondents, and odds ratios (ORs) were calculated using all other respondents as the reference group. Only significant ORs are reported in the text.

Statistical analyses were conducted using R, version 4.3.2 (R Project for Statistical Computing).^27^ Fisher exact, χ^2^, and Mann-Whitney tests involved R base functions. For ordinal logistic regressions, we used the package ordinal in R, version 2023.12-4.1.^28^ We assessed the proportional odds assumption for each ordinal regression by likelihood ratio tests. The distributions varied across each ordinal regression between symmetric or leptokurtic; thus, we used a logit link to construct the models. Binary regressions were conducted with the package MASS in R, version 7.3-65 using a binomial error distribution.^29^

When appropriate, Anderson-Darling tests, from the package nortest, version 1.0-4 in R,^30^ assessed normality, and Levene tests, from the package car, version 3.1.3 in R,^31^ assessed homoscedasticity of variance. Statistical significance was set at 2-sided α = .05, and Bonferroni correction was used for all multiple comparisons.

## Results

### Demographics and Participant Characteristics

There were 7648 consenting respondents, of whom 6612 completed the demographics section and 5123 completed the entire survey. (eTable 1 in Supplement 1 shows retention through sections.) Among the 6612 respondents (mean [SD] age, 24.3 [7.7] years), the sample was predominantly female (6217 [94.0%]); 308 (4.7%) were male. There were 67 respondents (1.0%) who identified as Aboriginal, 316 (4.8%) as Asian, 71 (1.1%) as Black or African American, 306 (4.6%) as Hispanic or Latino, 51 (0.8%) as Native American, 29 (0.4%) as Pacific Islander, 2 (0%) as Torres Strait Islander, 5438 (82.2%) as White, and 300 (4.5%) as other race or ethnicity, and 32 (0.5%) preferred not to say. Respondents represented 83 countries, with most residing in Australia (1981 [30.0%]), the UK (1409 [21.3%]), or the US (1195 [18.0%]) (Table).

**Table.** Demographic and Clinical Characteristics of Respondents

Characteristic	Eating disorder																					
	All (N = 6612), No. (%)	Undiagnosed (n = 2493)			AN (n = 1485)			AN plus BN (n = 425)			BN (n = 282)			BED (n = 261)			ARFID (n = 158)			OSFED (n = 214)		
		No. (%)	*z* Score	*P* value	No. (%)	*z* Score	*P* value	No. (%)	*z* Score	*P* value	No. (%)	*z* Score	*P* value	No. (%)	*z* Score	*P* value	No. (%)	*z* Score	*P* value	No. (%)	*z* Score	*P* value
**Demographic**																						
Age, mean (SD), y	24.3 (7.7)	23.8 (7.6)	NA	<.001	23.4 (6.9)	NA	<.001	23.7 (7.5)	NA	.33	25.1 (7.6)	NA	.03	30.1 (9.4)	NA	<.001	24.0 (7.6)	NA	1.00	26.5 (8.1)	NA	<.001
Sex																						
Female	6217 (94.0)	2268 (91.0)	−8.14	<.001	1432 (96.4)	4.44	<.001	417 (98.1)	3.68	.003	277 (98.2)	3.04	.009	238 (91.2)	−1.97	.008	149 (94.3)	0.15	.84	201 (93.9)	−0.06	.81
Intersex	26 (0.9)	8 (0.3)	−0.73		3 (0.2)	−1.33		1 (0.2)	−0.54		0	−1.08		3 (1.1)	1.99		0	−0.8		0	−0.93	
Male	308 (4.7)	185 (7.4)	8.29		39 (2.6)	−4.22		7 (1.6)	−3.05		2 (0.7)	−3.22		20 (7.7)	2.35		8 (5.1)	0.24		11 (5.1)	0.34	
Prefer not to say	61 (0.9)	32 (1.3)	2.34		11 (0.7)	−0.83		0	−2.06		3 (1.1)	0.25		0	−1.59		1 (0.6)	−0.39		2 (0.9)	0.02	
Gender identity																						
Man	524 (7.9)	280 (11.2)	7.74	<.001	74 (5.0)	−4.77	<.001	16 (3.8)	−3.28	<.001	12 (4.3)	−2.33	.002	23 (8.8)	0.54	.14	14 (8.9)	0.44	.18	19 (8.9)	0.52	.05
Nonbinary or gender-fluid	873 (13.2)	378 (15.2)	3.66		100 (6.7)	−4.88		35 (8.2)	−3.13		24 (8.5)	−2.38		23 (8.8)	−2.14		30 (19.0)	2.17		31 (14.5)	0.56	
Woman	5134 (77.6)	1797 (72.1)	−8.45		1259 (84.8)	7.49		370 (87.1)	4.81		246 (87.2)	3.95		213 (81.6)	1.57		113 (71.5)	−1.87		158 (73.8)	−1.36	
Different identity	46 (0.7)	24 (1.0)	2.03		5 (0.3)	−1.89		1 (0.2)	−1.18		0	−1.44		0	−1.38		0	−1.06		5 (2.3)	2.93	
Prefer not to say	35 (0.5)	14 (0.6)	0.28		7 (0.5)	−0.34		3 (0.7)	0.52		0	−1.25		2 (0.8)	0.54		1 (0.6)	0.18		1 (0.5)	−0.13	
Country of residence																						
Australia	1981 (30.0)	645 (25.9)	−5.59	<.001	490 (33.0)	2.9	<.001	135 (31.8)	0.84	.15	78 (27.7)	−0.86	.03	68 (26.1)	−1.41	.02	55 (34.8)	1.35	.32	69 (32.2)	0.74	.47
UK	1409 (21.3)	528 (21.1)	−0.20		362 (24.4)	3.28		105 (24.7)	1.77		74 (26.2)	2.07		50 (19.2)	−0.87		31 (19.6)	−0.52		52 (24.3)	1.09	
US	1195 (18.0)	406 (16.3)	−2.94		253 (17.0)	−1.18		77 (18.1)	0.02		33 (11.7)	−2.84		58 (22.2)	1.78		31 (19.6)	0.51		41 (19.2)	0.42	
Canada	686 (10.4)	294 (11.8)	2.94		123 (8.3)	−3		37 (8.7)	−1.17		32 (11.3)	0.55		31 (11.9)	0.81		13 (8.2)	−0.9		20 (9.3)	−0.5	
Ireland	303 (4.6)	137 (5.5)	2.76		72 (4.8)	0.56		16 (3.8)	−0.83		10 (3.5)	−0.85		8 (3.1)	−1.2		8 (5.1)	0.29		5 (2.3)	−1.6	
New Zealand	191 (2.9)	68 (2.7)	−0.61		48 (3.2)	0.9		10 (2.4)	−0.68		10 (3.5)	0.67		8 (3.1)	0.17		7 (4.4)	1.17		7 (3.3)	0.34	
Brazil	118 (1.8)	55 (2.2)	2.01		14 (0.9)	−2.78		6 (1.4)	−0.6		4 (1.4)	−0.47		11 (4.2)	3.03		4 (2.5)	0.72		2 (0.9)	−0.95	
Germany	94 (1.4)	31 (1.2)	−0.95		29 (2.0)	1.96		10 (2.4)	1.68		3 (1.1)	−0.52		1 (0.4)	−1.44		0	−1.52		4 (1.9)	0.56	
Other countries (n = 75)	635 (9.6)	328 (13.2)	7.63		94 (6.3)	−4.86		29 (6.8)	−2.01		38 (13.5)	2.26		26 (10.0)	0.2		9 (5.7)	−1.69		14 (6.5)	−1.54	
Race and ethnicity																						
Aboriginal	67 (1.0)	36 (1.4)	2.72	<.001	12 (0.8)	−0.9	<.001	4 (0.9)	−0.15	.03	1 (0.4)	−1.13	.39	1 (0.4)	−1.04	.41	1 (0.6)	−0.48	.86	3 (1.4)	0.58	.90
Asian	316 (4.8)	180 (7.2)	7.24		42 (2.8)	−4		8 (1.9)	−2.89		11 (3.9)	−0.71		12 (4.6)	−0.14		9 (5.7)	0.55		9 (4.2)	−0.4	
Black or African American	71 (1.1)	26 (1.0)	−0.19		11 (0.7)	−1.41		4 (0.9)	−0.27		5 (1.8)	1.16		5 (1.9)	1.35		3 (1.9)	1.02		2 (0.9)	−0.2	
Hispanic or Latino	306 (4.6)	120 (4.8)	0.56		48 (3.2)	−2.91		18 (4.2)	−0.4		16 (5.7)	0.85		13 (5.0)	0.28		9 (5.7)	0.65		10 (4.7)	0.03	
American Indian or Alaska Native	51 (0.8)	18 (0.7)	−0.36		5 (0.3)	−2.17		3 (0.7)	−0.16		0	−1.51		3 (1.1)	0.71		0	−1.12		3 (1.4)	1.07	
Pacific Islander	29 (0.4)	17 (0.7)	2.33		1 (0)	−1.57		0	−1.41		2 (0.7)	0.70		0	−1.09		0	−0.84		1 (0.5)	0.06	
Torres Strait Islander	2 (0)	0	−1.1		1 (0)	0.93		1 (0.2)	2.51		0	−0.30		0	−0.29		0	−0.22		0	−0.26	
White	5438 (82.2)	1947 (78.1)	−6.8		1300 (87.5)	6.07		368 (86.6)	2.42		230 (81.6)	−0.31		217 (83.1)	0.39		130 (82.3)	0.01		180 (84.1)	0.73	
Other^a^	300 (4.5)	138 (5.5)	3.03		56 (3.8)	−1.61		16 (3.8)	−0.79		17 (6.0)	1.23		7 (2.7)	−1.47		5 (3.2)	−0.84		5 (2.3)	−1.57	
Prefer not to say	32 (0.5)	10 (0.4)	−0.76		7 (0.5)	−0.08		3 (0.7)	0.68		0	−1.20		3 (1.1)	1.58		1 (0.6)	0.27		1 (0.5)	−0.04	
Employment																						
Full-time	1916 (29.0)	720 (28.9)	−0.13	<.001	360 (24.2)	−4.57	<.001	110 (25.9)	−1.45	.35	95 (33.7)	1.78	.65	125 (47.9)	6.87	<.001	43 (27.2)	−0.49	.18	71 (33.2)	1.38	.002
Part-time	1615 (24.4)	611 (24.5)	0.12		390 (26.3)	1.87		106 (24.9)	0.26		65 (23.0)	−0.55		50 (19.2)	−2.02		38 (24.1)	−0.11		37 (17.3)	−2.47	
Home duties	148 (2.2)	61 (2.4)	0.89		26 (1.8)	−1.44		8 (1.9)	−0.51		6 (2.1)	−0.13		9 (3.4)	1.35		6 (3.8)	1.34		5 (2.3)	0.10	
Unemployed	645 (9.8)	260 (10.4)	1.44		121 (8.1)	−2.37		40 (9.4)	0.25		25 (8.9)	−0.51		22 (8.4)	−0.74		23 (14.6)	2.06		15 (7.0)	−1.38	
Retired	17 (0.3)	5 (0.2)	−0.71		3 (0.2)	−0.48		0	−1.08		0	−0.87		3 (1.1)	2.9		0	−0.65		1 (0.5)	0.62	
Disability pension	240 (3.6)	48 (1.9)	−5.76		64 (4.3)	1.59		19 (4.5)	0.96		9 (3.2)	−0.4		8 (3.1)	−0.5		9 (5.7)	1.41		18 (8.4)	3.80	
Student	1897 (28.7)	734 (29.4)	1.05		496 (33.4)	4.56		128 (30.1)	0.67		79 (28.0)	−0.26		38 (14.6)	−5.15		36 (22.8)	−1.66		62 (29.0)	0.09	
Other	134 (2.0)	54 (2.2)	0.63		25 (1.7)	−1.07		14 (3.3)	1.92		3 (1.1)	−1.17		6 (2.3)	0.32		3 (1.9)	−0.12		5 (2.3)	0.33	
Educational level																						
Elementary or primary school	49 (0.7)	20 (0.8)	0.45	.02	12 (0.8)	0.34	.41	1 (0.2)	−1.26	.24	1 (0.4)	−0.77	.63	1 (0.4)	−0.69	<.001	1 (0.6)	−0.16	.28	2 (0.9)	0.34	.01
Secondary or high school	2572 (38.9)	1032 (41.4)	3.24		592 (39.9)	0.87		165 (38.8)	−0.03		102 (36.2)	0.23		53 (20.3)	−6.29		70 (44.3)	1.41		61 (28.5)	−3.17	
Trade or vocational training	1061 (16.0)	399 (16.0)	−0.07		212 (14.3)	−2.11		66 (15.5)	−0.3		44 (15.6)	−0.21		53 (20.3)	1.91		27 (17.1)	0.36		37 (17.3)	0.5	
Undergraduate university	2073 (31.4)	749 (30.0)	−1.73		472 (31.8)	0.41		147 (34.6)	1.49		90 (31.9)	0.21		101 (38.7)	2.61		49 (31.0)	−0.09		74 (34.6)	1.03	
Postgraduate university	707 (10.7)	242 (9.7)	−1.99		159 (10.7)	0.04		34 (8.0)	−1.85		38 (13.5)	1.55		48 (18.4)	4.12		8 (5.1)	−2.31		36 (16.8)	2.96	
Other	150 (2.3)	50 (2.0)	−1.18		38 (2.6)	0.81		12 (2.8)	0.77		7 (2.5)	0.23		5 (1.9)	−0.41		3 (1.9)	−0.33		4 (1.9)	−0.41	
**Clinical **																						
Comorbidities^b^	6241 (94.4)	2288 (91.8)	−7.18	<.001	1417 (95.4)	1.96	.05	408 (96.0)	1.49	.14	269 (95.4)	0.75	.46	246 (94.3)	1.94	.92	157 (99.4)	2.75	.006	205 (95.8)	0.91	.36
Depression	4333 (65.5)	1511 (60.6)	−6.53	<.001	1002 (67.5)	1.8	.07	305 (71.8)	2.80	.005	172 (61.0)	−1.63	.10	174 (66.7)	0.4	.69	110 (69.6)	1.1	.27	142 (66.4)	0.26	.79
GAD	3579 (54.1)	1184 (47.5)	−8.41	<.001	877 (59.1)	4.34	<.001	241 (56.7)	1.11	.27	121 (42.9)	−3.86	<.001	138 (52.9)	−0.41	.68	97 (61.4)	1.86	.06	128 (59.8)	1.7	.09
Body dysmorphic disorder	2614 (39.5)	810 (32.5)	−9.09	<.001	637 (42.9)	3.02	.003	220 (51.8)	5.34	<.001	116 (41.1)	0.57	.57	61 (23.4)	−5.44	<.001	36 (22.8)	−4.35	<.001	91 (42.5)	0.91	.36
ADHD	2181 (33.0)	873 (35.0)	2.76	.006	350 (23.6)	−8.75	<.001	129 (30.4)	−1.19	.24	80 (28.4)	−1.68	.09	95 (36.4)	1.2	.23	73 (46.2)	3.58	<.001	79 (36.9)	1.25	.21
SAD	2057 (31.1)	776 (31.1)	0.04	.97	427 (28.8)	−2.21	.03	133 (31.3)	0.09	.93	65 (23.0)	−2.98	.003	76 (29.1)	−0.7	.48	53 (33.5)	0.67	.50	55 (25.7)	−1.73	.08
PTSD	1494 (22.6)	492 (19.7)	−4.28	<.001	315 (21.2)	−1.42	.16	112 (26.4)	1.93	.05	53 (18.8)	−1.69	.09	63 (24.1)	0.62	.54	44 (27.8)	1.61	.11	51 (23.8)	0.45	.65
ASD	1473 (22.3)	573 (23.0)	1.10	.27	279 (18.8)	−3.66	<.001	78 (18.4)	−2.00	.05	35 (12.4)	−4.06	<.001	45 (17.2)	−1.99	.05	62 (39.2)	5.19	<.001	49 (22.9)	0.23	.82
Complex PTSD	1370 (20.7)	371 (14.9)	−9.09	<.001	309 (20.8)	0.11	.91	97 (22.8)	1.11	.27	43 (15.2)	−2.31	.02	51 (19.5)	−0.47	.64	47 (29.7)	2.84	.005	72 (33.6)	4.75	<.001
OCD	1269 (19.2)	353 (14.2)	−8.06	<.001	329 (22.2)	3.31	<.001	79 (18.6)	−0.32	.75	48 (17.0)	−0.94	.35	47 (18.0)	−0.49	.62	33 (20.9)	0.55	.58	45 (21.0)	0.7	.48
Emotional personality disorders	1261 (19.1)	375 (15.0)	−6.47	<.001	262 (17.6)	−1.57	.12	101 (23.8)	2.55	.01	72 (25.5)	2.83	.005	48 (18.4)	−0.27	.78	35 (22.2)	1.00	.32	44 (20.6)	0.57	.57
Drug use or dependence	1006 (15.2)	447 (17.9)	4.81	<.001	144 (9.7)	−6.71	<.001	67 (15.8)	0.34	.74	38 (13.5)	−0.82	.41	26 (10.0)	−2.4	.02	34 (21.5)	2.24	.03	26 (12.1)	−1.26	.21
Panic disorder	789 (11.9)	279 (11.2)	−1.41	.16	155 (10.4)	−2.00	.05	54 (12.7)	0.52	.60	23 (8.2)	−1.99	.05	26 (10.0)	−1.00	.32	35 (22.2)	4.02	<.001	22 (10.3)	−0.75	.45
Alcohol use or dependence	613 (9.3)	233 (9.3)	0.20	.84	104 (7.0)	−3.41	<.001	61 (14.4)	3.75	<.001	31 (11.0)	1.03	.30	10 (3.8)	−3.09	.002	10 (6.3)	−1.28	.20	14 (6.5)	−1.39	.16
Bipolar disorder	602 (9.1)	182 (7.3)	−3.94	<.001	114 (7.7)	−2.15	.03	53 (12.5)	2.51	.01	28 (9.9)	0.50	.62	24 (9.2)	0.06	.95	15 (9.5)	0.18	.86	16 (7.5)	−0.83	.40
Dissociative disorders	221 (3.3)	85 (3.4)	0.29	.77	37 (2.5)	−2.04	.04	9 (2.1)	−1.44	.15	7 (2.5)	−0.81	.42	5 (1.9)	−1.30	.19	10 (6.3)	2.13	.03	10 (4.7)	1.12	.27
Schizophrenia spectrum, other psychotic disorders	119 (1.8)	43 (1.7)	−0.29	.78	19 (1.3)	−1.67	.10	14 (3.3)	2.43	.02	7 (2.5)	0.90	.37	1 (0.4)	−1.74	.09	3 (1.9)	0.11	.91	2 (0.9)	−0.95	.34
Anxious personality disorders	26 (0.4)	5 (0.2)	NA	NA	6 (0.4)	NA	NA	2 (0.5)	NA	NA	2 (0.7)	NA	NA	0	NA	NA	0	NA	NA	2 (0.9)	NA	NA
Gender dysphoria	20 (0.3)	7 (0.3)	NA	NA	5 (0.3)	NA	NA	0	NA	NA	0	NA	NA	0	NA	NA	2 (1.3)	NA	NA	0	NA	NA
Paranoid personality disorders	10 (0.2)	5 (0.2)	NA	NA	2 (0.1)	NA	NA	1 (0.2)	NA	NA	0	NA	NA	0	NA	NA	0	NA	NA	1 (0.5)	NA	NA
BFRB	8 (0.1)	1 (0)	NA	NA	1 (0.1)	NA	NA	0	NA	NA	1 (0.4)	NA	NA	0	NA	NA	0	NA	NA	0	NA	NA
Other	106 (1.6)	NA	NA	NA	NA	NA	NA	NA	NA	NA	NA	NA	NA	NA	NA	NA	NA	NA	NA	NA	NA	NA
Current psychological intervention	5770 (87.3)	1833 (73.5)	−26.07	<.001	1434 (96.6)	12.21	<.001	416 (97.9)	6.79	<.001	262 (92.9)	2.90	.004	238 (91.2)	1.94	.05	146 (92.4)	1.96	.05	201 (94.0)	2.97	.003
Ever hospitalized	1700 (25.7)	63 (2.5)	−33.56	<.001	785 (52.9)	27.19	<.001	212 (49.9)	11.79	<.001	59 (20.9)	−1.88	.06	12 (4.6)	−7.96	<.001	30 (19.0)	−1.96	.05	40 (18.7)	−2.39	.01
Prescribed status, No./total No. (%)																						
Ever prescribed medication	4531/6136 (73.8)	1338/2358 (56.7)	−24.08	<.001	1157/1376 (84.1)	9.81	<.001	332/388 (85.6)	5.43	<.001	220/260 (84.6)	4.04	<.001	208/242 (86.0)	4.07	<.001	109/139 (78.4)	1.24	.22	169/194 (87.1)	3.28	.001
Currently prescribed medication	3122/6136 (50.9)	852/2358 (36.1)	−4.89	<.001	851/1376 (61.8)	3.93	<.001	218/388 (56.2)	−1.31	.19	148/260 (56.9)	−0.53	.60	154/242 (63.6)	1.87	.06	75/139 (54.0)	−0.01	.99	125/194 (64.4)	2.19	.03
Self-reported substance issue, No./total No. (%)	2863/5804 (49.3)	111/22 230 (49.9)	0.73	.48	593/1303 (45.5)	−3.13	.002	199/367 (54.2)	1.94	.06	129/247 (52.2)	0.93	.38	89/230 (38.7)	−3.29	.001	79/131 (60.3)	2.54	.01	78/185 (42.2)	−1.83	.08

Abbreviations: ADHD, attention-deficit/hyperactivity disorder; AN, anorexia nervosa; ARFID, avoidant/restrictive food intake disorder; ASD, autism spectrum disorder; BED, binge-eating disorder; BFRB, body-focused repetitive behavior; BN, bulimia nervosa; GAD, generalized anxiety disorder; LSD, lysergic acid diethylamide; NA, not applicable; OCD, obsessive-compulsive disorder; OSFED, other specified feeding or ED; PTSD, posttraumatic stress disorder; SAD, social anxiety disorder.

^a^ Includes individuals who selected the other category from a checklist. Respondents provided additional details in an open-text field, which yielded the following categories: Black other, Indian, Inuit, Jewish, Métis, Middle Eastern, multiracial, Romani, White/Aboriginal, White/Asian, White/Black, White/Jewish, White/Latin American, White/Middle Eastern, White/Pacific Islander, White/Romani, or other.

^b^ Percentages do not sum to 100% because most respondents reported multiple comorbidities.

A clinically diagnosed ED was reported by 4119 (62.3%) of respondents, while 2493 (37.7%) reported disordered eating causing significant distress (undiagnosed category). Of the diagnosed EDs, 2696 (40.8%) were AN, 1258 (19.0%) were BN, 757 (11.4%) were BED, 589 (8.9%) were ARFID, 581 (8.8%) were OSFED, 257 (3.9%) were unspecified feeding or an ED, 48 (0.7%) were rumination disorder, and 45 (0.7%) were pica. Many respondents self-reported multiple ED diagnoses with 90 different types of ED combinations reported (eTable 2 in Supplement 1).

Demographic differences across diagnoses were apparent (Table). Respondents with OSFED (mean [SD] age, 26.5 [8.1] years; *P* < .001) and BED (mean [D] age, 30.1 [9.4] years; *P* < .001) were significantly older than those with other EDs (AN: mean [SD] age, 23.4 [6.9] years; *P* < .001), while respondents with AN plus BN (*z* score = 3.68; *P* = .003) and AN alone (*z* score = 4.44; *P* < .001) were more likely to be female. Respondents who had BED (*z* score = 2.35; *P* = .008) and were undiagnosed (*z* score = 8.29; *P* < .001) were more likely to be male. Asian ethnicity was overrepresented among respondents who were undiagnosed (*z* score = 7.24; *P* < .001). BED was associated with full-time employment (*z* score = 6.87; *P* < .001) and AN with being a student (*z* score = 4.56; *P* < .001).

Respondents reported a high prevalence of current psychological interventions (5770 of 6612 [87.3%]), prescription psychotropic use (3122 of 6136 [50.9%]), and hospitalizations (1700 of 6612 [25.7%]) (Table). Unsurprisingly, respondents in the undiagnosed category were less likely to be receiving psychological interventions or a prescribed medication. Those with AN or AN plus BN reported more hospitalizations than all the ED types.

Among the 6612 respondents who completed the demographics section, common comorbid conditions (Table) included depression (4333 [65.5%]), generalized anxiety disorder (3579 [54.1%]), body dysmorphic disorder (2614 [39.5%]), attention-deficit/hyperactivity disorder (2181 [33.0%]), and social anxiety disorder (2057 [31.1%]) (Table). Comorbidities were more likely in those with ARFID. Problematic substance use was self-reported by many (2863 of 5804 [49.3%]) but less with those with BED and AN and more with those with ARFID. In the cohort, 1006 (15.2%) self-reported a comorbid drug dependence, and 613 (9.3%) reported alcohol use or dependence.

### Drug Use

The most commonly used drugs among respondents in the past 12 months were caffeine (5676 of 5864 [96.8%]), alcohol (4759 of 5722 [83.2%]), cannabis products (3018 of 5383 [56.1%]), and nicotine (2669 of 5604 [47.6%]). Percentages reflect the proportion of people providing yes or no responses for recent use of the drug in each relevant survey section.

**Figure 1.**
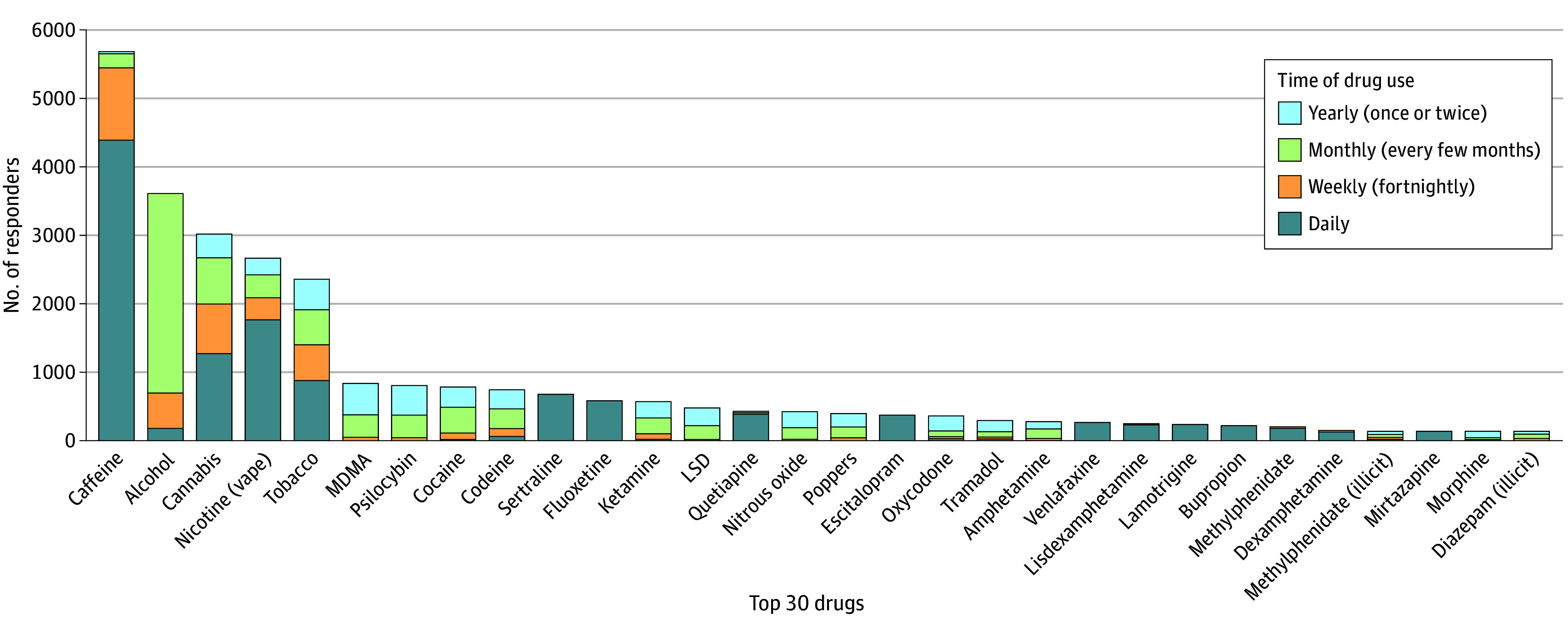
Top 30 Most Commonly Used Drugs by Frequency of Use LSD indicates lysergic acid diethylamide; MDMA, 3,4-methylenedioxymethamphetamine (or ecstasy).

Individuals tended to use caffeine, nicotine, tobacco, and prescription drugs daily (Figure 1). Daily caffeine use was more frequent in those with AN (OR, 1.46 [95% CI, 1.24-1.71]; *P* < .001) and AN plus BN (OR, 1.80 [95% CI, 1.35-2.46]; *P* < .001). Daily tobacco use was more likely with those with AN plus BN (OR, 1.40 [95% CI, 1.03-1.78]; *P* = .03), and daily nicotine use was more likely with those with ARFID (OR, 1.51 [95% CI, 1.05-2.15]; *P* = .02).

A small proportion of respondents who used alcohol consumed alcohol daily (178 of 3611 [4.9%]); monthly use of alcohol was more common (1053 of 3611 [29.2%]). Daily alcohol use was more likely among respondents with AN plus BN (OR, 1.74 [95% CI, 1.02-2.79]; *P* = .03) and with BN (OR, 2.71 [95% CI, 1.58-4.38]; *P* < .001).

Daily cannabis use was common (1272 of 3018 [42.1%]), particularly among those who had ARFID (OR, 2.4 [95% CI, 1.65-3.46]; *P* < .001) and those who were undiagnosed (OR, 1.21 [95% CI, 1.06-1.38]; *P* < .001). MDMA (839 of 5201 [16.1%]), psilocybin (807 of 5247 [15.4%]), ketamine (573 of 5201 [11.0%]), and cocaine (787 of 5276 [14.9%]) were commonly used but typically monthly or less.

Commonly used prescription medications among 6136 respondents included the selective serotonin reuptake inhibitors (SSRIs) sertraline (678 [11.0%]), fluoxetine (582 [9.5%]), and escitalopram (488 [8.0%]); the atypical antipsychotic quetiapine (436 [7.1%]); the serotonin-norepinephrine reuptake inhibitor antidepressant venlafaxine (267 [4.4%]); the mood stabilizer lamotrigine (241 [3.9%]); the stimulant lisdexamfetamine (235 [3.8%]); and the antidepressant bupropion (224 [3.7%]). Among 5184 respondents, use of opioid analgesic drugs, including codeine (748 [14.4%]), oxycodone (366 [7.1%]), tramadol (297 [5.7%]), and morphine (140 [2.7%]), was also reported, with daily use uncommon.

### Self-Reported Efficacy of Drugs for ED Symptoms

**Figure 2.**
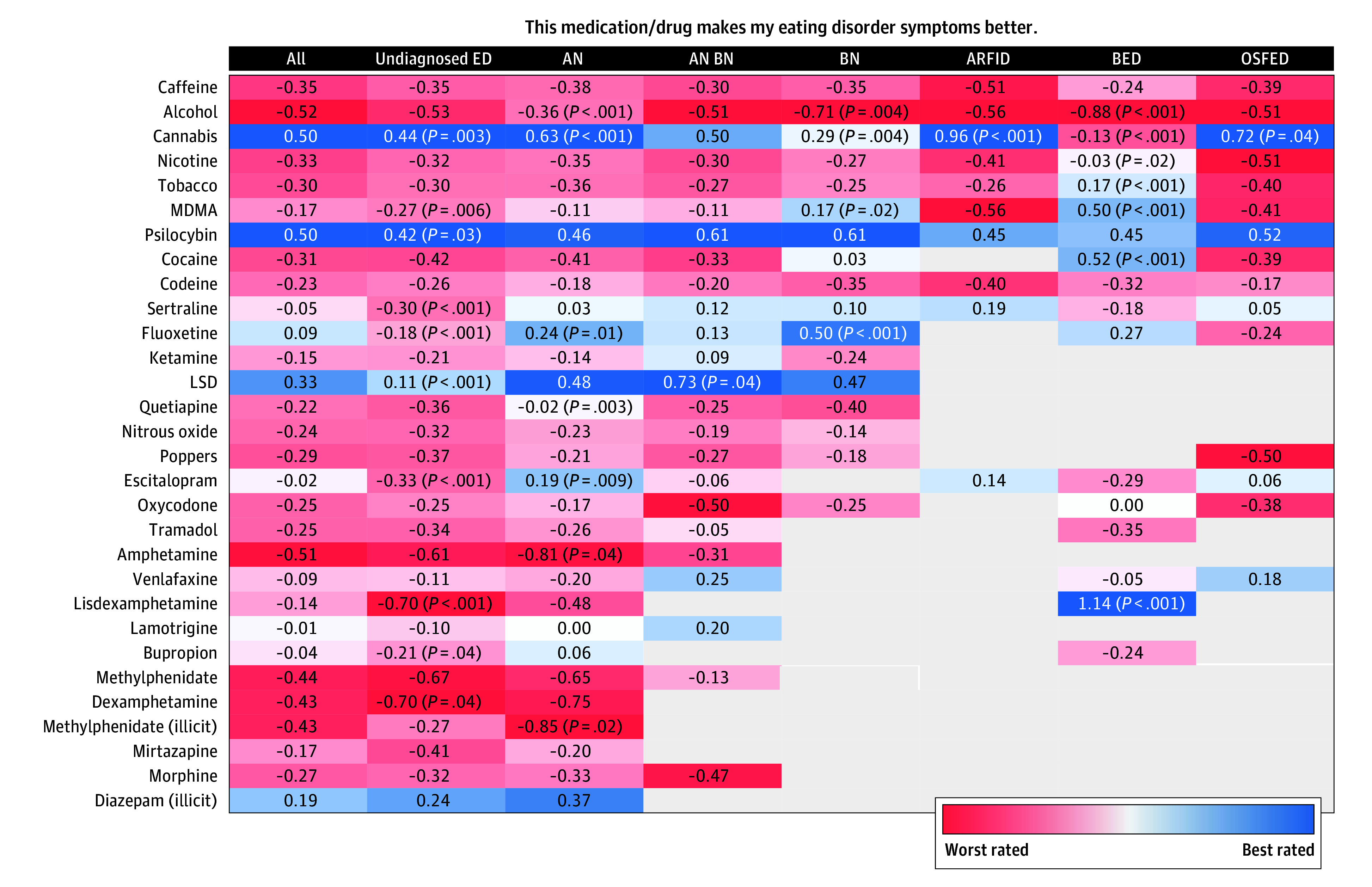
Mean Ratings of Top 30 Drugs for Improving Eating Disorder (ED) Symptoms Responses to the question presented were measured on a 5-point Likert scale (−2, strongly disagree; −1, disagree; 0, neutral; 1, agree; and 2, strongly agree). Only cells with more than 15 responses are shown; otherwise, the cell is empty. Mann-Whitney tests were used to determine whether the distribution of Likert responses differed significantly between a given diagnosis group and the other respondents. AN indicates anorexia nervosa; ARFID, avoidant/restrictive food intake disorder; BED, binge-eating disorder; BN, bulimia nervosa; LSD, lysergic acid diethylamide; MDMA, 3,4-methylenedioxymethamphetamine (or ecstasy); OSFED, other specified feeding or ED.

Overall, the best-rated drugs for ED symptoms among respondents were psilocybin (mean rating, 0.50; 807 of 5247 [15.4%]), cannabis (mean rating, 0.50; 3018 of 5383 [56.1%]), and lysergic acid diethylamide (LSD; mean rating, 0.33; 487 of 5247 [9.3%]). Percentages reflect the overall proportion of survey respondents asserting recent use of that drug (Figure 2).

Among respondents’ reporting of psychotropic medications, illicitly sourced diazepam (mean rating, 0.19; 136 of 5123 [2.7%]) and prescribed fluoxetine (mean rating, 0.09; 582 of 6136 [9.5%]) received small positive ratings. Cocaine (mean rating, −0.31; 787 of 5277 [14.9%]), MDMA (mean rating, −0.17; 839 of 5201 [16.1%]), and ketamine (mean rating, −0.15; 574 of 5201 [11.0%]) were negatively rated. Alcohol (mean rating, −0.52; 4759 of 5722 [83.2%]) and amphetamines (mean rating, −0.52; 281 of 5276 [5.3%]) received the poorest ratings.

For respondents with AN, cannabis (mean rating, 0.63; 595 of 1234 [48.2%]) received the highest rating, followed by LSD (mean rating, 0.48; 83 of 1202 [6.9%]), psilocybin (mean rating, 0.46; 131 of 1202 respondents [10.9%]), and illicitly sourced diazepam (mean rating, 0.37; 40 of 1173 respondents [3.4%]). A diagnosis of AN increased the likelihood that cannabis improved ED symptoms (OR, 1.38 [95% CI, 1.17-1.62]; *P* < .001). Similarly with respondents who were undiagnosed, cannabis (mean rating, 0.44; 1222 of 2031 [60.2%]), psilocybin (mean rating, 0.42; 337 of 1977 [17.0%]), and illicitly sourced diazepam (mean rating, 0.24; 42 of 1923 [2.2%]) rated highly.

Respondents with ARFID rated cannabis highly (mean rating, 0.96; 78 of 122 [63.9%]), and an ARFID diagnosis was associated with a high cannabis rating (OR, 2.37 [95% CI, 1.60-3.53]; *P* < .001). In respondents with ARFID, psilocybin (mean rating, 0.45; 20 of 120 [16.7%]) was also favorably rated.

For respondents with BN, psilocybin (mean rating, 0.61; 33 of 216 [15.3%]) and fluoxetine (mean rating, 0.50; 36 of 260 [13.8%]) were highly rated, more so than in the overall cohort (OR, 3.78 [95% CI, 1.68-6.04]; *P* < .001). Quetiapine was poorly rated among respondents with BN (mean rating, −0.40; 15 of 260 [5.8%]) and those with AN plus BN (mean rating, −0.25; 32 of 388 [8.2%]).

Respondents with a diagnosis of AN plus BN reported LSD (mean rating, 0.73; 30 of 317 [9.5%]) followed by psilocybin (mean rating, 0.60; 56 of 317 [17.7%]) as best for ED symptoms. A diagnosis of AN plus BN was associated with a positive rating for LSD (OR, 2.18 [95% CI, 1.07-4.50]; *P* = .03).

For BED, lisdexamfetamine was highly rated among respondents (mean rating, 1.14; 23 of 242 [9.5%]) as was cocaine (mean rating, 0.50; 23 of 208 [11.1%]) and MDMA (mean rating, 0.50; 24 of 205 [11.7%]). Respondents with BED were more likely than the overall population to highly rate lisdexamfetamine (OR, 7.74 [95% CI, 3.79-16.27]; *P* < .001), cocaine (OR, 3.94 [95% CI, 1.85-8.45]; *P* < .001), and MDMA (OR, 3.56 [95% CI, 1.74-6.89]; *P* < .001).

When asked to identify their drug of choice for self-medicating ED symptoms, the most popular drug among respondents was cannabis. When normalized by the number of users of that drug, the top drug was fluoxetine (eTable 3 in Supplement 1).

### Self-Reported Efficacy of Drugs for Overall Mental Health

**Figure 3.**
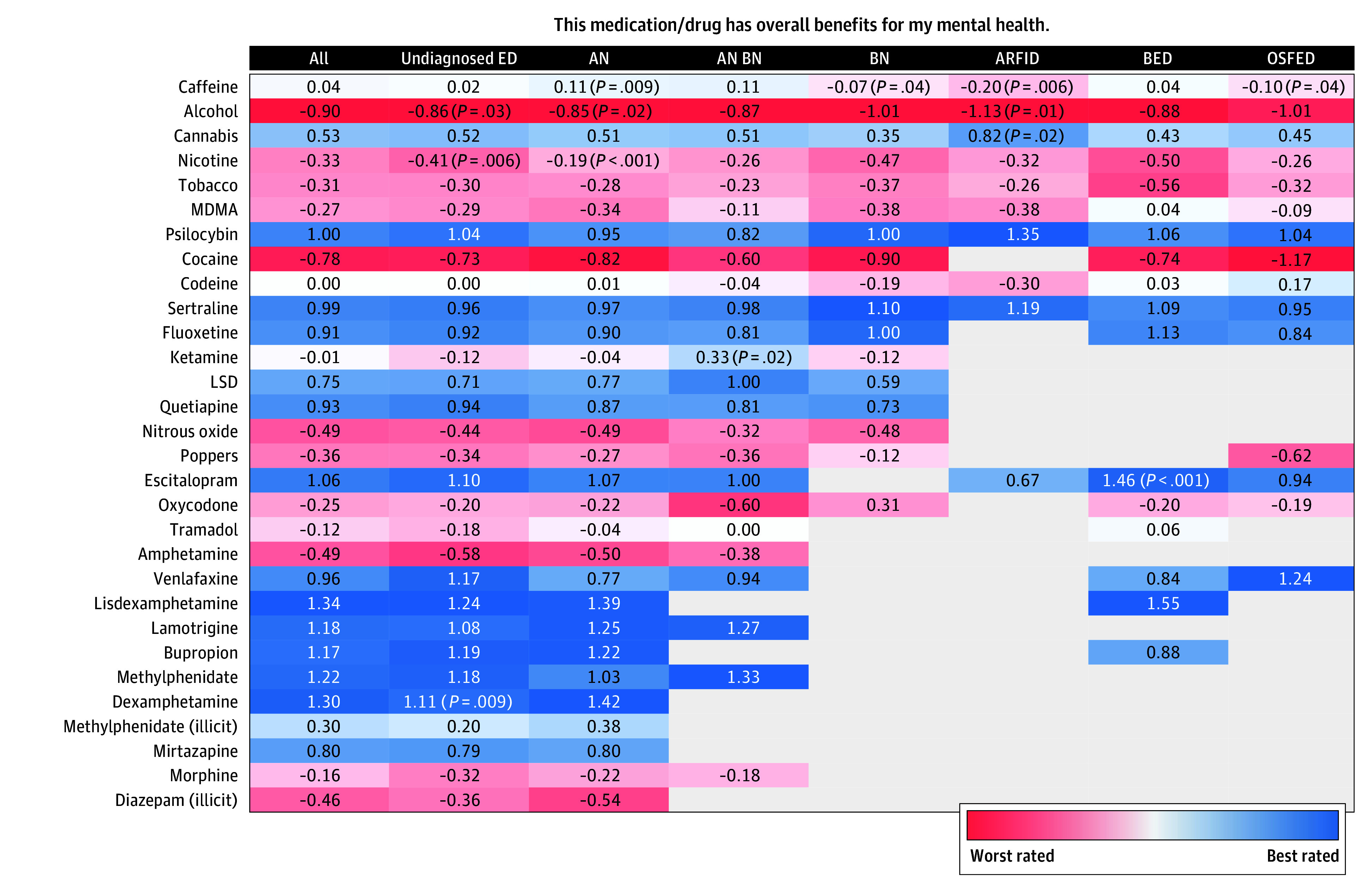
Mean Ratings of Top 30 Drugs for Overall Mental Health Responses to the question presented were measured on a 5-point Likert scale (−2, strongly disagree; −1, disagree; 0, neutral; 1, agree; and 2, strongly agree). Only cells with more than 15 responses are shown; otherwise, the cell is empty. Mann-Whitney tests were used to determine whether the distribution of Likert responses differed significantly between a given diagnosis group and the other respondents. AN indicates anorexia nervosa; ARFID, avoidant/restrictive food intake disorder; BED, binge-eating disorder; BN, bulimia nervosa; ED, eating disorder; LSD, lysergic acid diethylamide; MDMA, 3,4-methylenedioxymethamphetamine (or ecstasy); OSFED, other specified feeding or ED.

Overall, among 6136 respondents, the best-rated drugs for mental health were prescription psychotropics, including lisdexamfetamine (mean rating, 1.34; 209 [3.4%]), methylphenidate (mean rating, 1.21; 204 [3.3%]), lamotrigine (mean rating, 1.19; 237 [3.9%]), bupropion (mean rating, 1.18; 216 [3.5%]), and escitalopram (mean rating, 1.06; 368 [6.0%]). Psilocybin (mean rating, 1.00; 807 of 5247 [15.4%]), LSD (mean rating, 0.75; 487 of 5247 [9.3%]), and cannabis (mean rating, 0.57; 3009 of 5383 [55.9%]) also rated highly (Figure 3).

Lisdexamfetamine was highly rated across all EDs and was the best-rated drug among respondents who were undiagnosed (mean rating, 1.23; 68 of 2358 [2.9%]) and those with BED (mean rating, 1.55; 23 of 260 [8.8%]). Escitalopram was rated positively by respondents for BED (mean rating, 1.46; 24 of 242 [9.9%]), more so than in other EDs (OR, 3.08 [95% CI, 1.38-7.05]; *P* = .006). Fluoxetine was highly rated for BN (mean rating, 1.00; 36 of 260 [13.8%]). Sertraline (mean rating, 1.19; 16 of 139 [11.5%]) and psilocybin (mean rating, 1.35; 20 of 120 [16.7%]) were highly rated by respondents with ARFID, more so than by those with other conditions (OR, 1.57 [95% CI, 1.06-2.34]; *P* = .03).

Alcohol was the worst-rated drug for mental health (mean rating, −0.90; 4759 of 5722 [83.2%]), with poor ratings also evident for cocaine (mean rating, −0.78; 787 of 5276 [14.9%]), MDMA (mean rating, −0.27; 840 of 5201 [16.2%]), and amphetamines (mean rating, −0.49; 281 of 5276 [5.3%]). Ketamine (mean rating, −0.01; 574 of 5201 [11.0%]) was rated as neutral for overall mental health, except for AN plus BN (mean rating, 0.33; 45 of 388 [11.6%]), with which respondents were more likely to report a favorable rating (OR, 1.91 [95% CI, 1.09-3.34]; *P* = .02).

### Self-Reported Tolerability of Drugs

**Figure 4.**
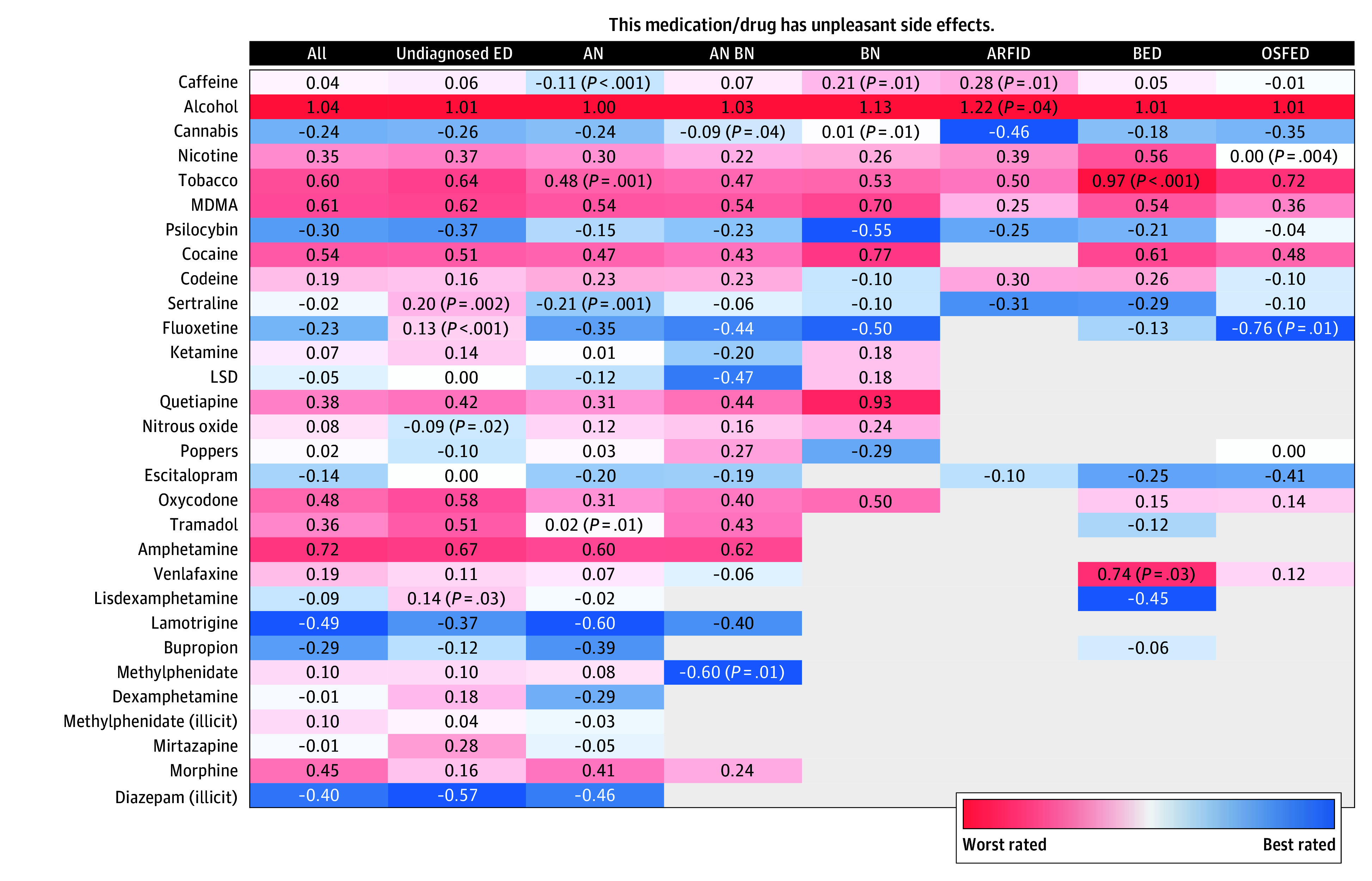
Mean Ratings of Top 30 Drugs for Tolerability Responses to the question presented were measured on a 5-point Likert scale (−2, strongly disagree; −1, disagree; 0, neutral; 1, agree; and 2, strongly agree). Only cells with more than 15 responses are shown; otherwise, the cell is empty. Mann-Whitney tests were used to determine whether the distribution of Likert responses differed significantly between a given diagnosis group and the remaining respondents. AN indicates anorexia nervosa; ARFID, avoidant/restrictive food intake disorder; BED, binge-eating disorder; BN, bulimia nervosa; ED, eating disorder; LSD, lysergic acid diethylamide; MDMA, 3,4-methylenedioxymethamphetamine (or ecstasy); OSFED, other specified feeding or ED.

Overall, unpleasant adverse effects among respondents were least likely to be reported with lamotrigine (mean rating, −0.49; 237 of 6136 [3.9%]), illicitly sourced diazepam (mean rating, −0.40; 139 of 5123 [2.7%]), bupropion (mean rating, −0.29; 209 of 6136 [3.4%]), and psilocybin (mean rating, −0.30; 807 of 5247 [15.4%]) (Figure 4). Mean ratings among respondents for alcohol (1.04; 4759 of 5722 [83.2%]), tobacco (0.60; 2359 of 5201 [45.4%]), nicotine (0.35; 2669 of 5604 [47.6%]), cocaine (0.54; 787 of 5724 [13.7%]), MDMA (0.61; 840 of 5724 [14.7%]), quetiapine (0.38; 435 of 5724 [7.6%]), amphetamine (0.72; 281 of 5724 [4.9%]), and oxycodone (0.48; 366 of 5724 [6.4%]) were poor. Ketamine attracted neutral mean ratings (0.07; 573 of 5201 respondents [11.0%]).

Respondents who were undiagnosed gave good mean ratings for illicitly sourced diazepam (−0.57; 42 of 1923 [2.2%]), lamotrigine (−0.37; 51 of 2358 [2.2%]), and psilocybin (−0.37; 337 of 1977 [17.0%]). Individuals with AN assigned mean ratings for lamotrigine (−0.60; 78 of 1376 [5.7%]) and bupropion (−0.39; 35 of 1376 [2.5%] as the least problematic and were less likely to report adverse effects with sertraline (OR, 0.62 [95% CI, 0.46-0.83]; *P* = .001). In respondents with BN, psilocybin (mean rating, −0.55; 33 of 216 [15.3%]) and fluoxetine (mean rating, −0.50; 36 of 260 [13.8%]) rated well.

For 242 respondents with BED, mean ratings for lisdexamfetamine (−0.45; 23 [9.5%]) and sertraline (−0.29; 34 [14.0%]) were the best. Individuals with BED were also twice as likely to report negative effects with venlafaxine (OR, 2.83 [95% CI, 1.19-6.81]; *P* = .02). Individuals with OSFED favored fluoxetine (mean rating, −0.76; 24 of 194 [12.4%]), while in respondents with ARFID, sertraline (mean rating, −0.31; 16 of 139 [11.5%]) and psilocybin (mean rating, −0.25; 20 of 120 [16.7%]) were favored. When asked whether they had a problem with a specific drug, nicotine, tobacco, cannabis, alcohol, and caffeine received the most affirmative responses, particularly in daily users of these drugs, and alcohol, nicotine, and tobacco were rated as the most harmful drugs (eTable 4 in Supplement 1).

## Discussion

The MED-FED survey provides unique insights into the lived experience of people with EDs around their prescribed and nonprescribed drug use. The survey attracted a large, international cohort representing all major diagnosable EDs. The many respondents with undiagnosed EDs underline previous observations that EDs often go unrecognized, leading to insufficient access to psychological and pharmacologic interventions.^32^

A striking outcome was the favorable self-reported ratings of psychedelics and cannabis for alleviating ED symptoms, eclipsing the ratings of commonly prescribed psychotropics. The only exception was the positive rating of 2 US Food and Drug Administration–approved drugs—lisdexamfetamine for treating BED and fluoxetine for treating BN, conditions for which they are specifically indicated.^2^

There was widespread use of the SSRI antidepressants sertraline, fluoxetine, and escitalopram and the atypical antipsychotic quetiapine, consistent with previous reports.^33,34^ The SSRIs and bupropion were rated favorably in terms of their effects on general mental health and tolerability.^2^ Quetiapine was rated favorably for mental health but with poor tolerability, presumably due to its sedative and metabolic effects.^35,36,37^ Venlafaxine also had poor tolerability, particularly in those with BED, reflecting its more burdensome adverse effect profile compared with SSRIs.^38^

Lisdexamfetamine rated well for overall mental health among respondents with BED and AN and among those who were undiagnosed, perhaps reflecting the association between attention-deficit/hyperactivity disorder and EDs.^39,40^ While highly rated for ED symptoms among respondents with BED, lisdexamfetamine was very poorly rated among respondents who were undiagnosed and those with AN, presumably due to its anorectic effects. The prominent use of lamotrigine presumably reflects its approved use for treating bipolar depression^41^ and comorbid bipolar disorder reported in this population. Lamotrigine scored well for overall mental health and also for tolerability.

Cannabis users, many of them daily users, reported perceived benefits for their ED symptoms. There is scant research around the use of cannabinoids in individuals with EDs apart from small trials supporting the efficacy of dronabinol (synthetic tetrahydrocannabinol) in those with AN.^21^ Cannabis may benefit people with restrictive and food-aversive EDs, like AN and ARFID, by increasing the hedonic value of food.^42,43^ In contrast, cannabis was poorly rated in individuals with BN and BED, likely due to the appetite-enhancing effects, which may increase binge-and-purge episodes, exacerbating ED psychopathology.^44^

Psilocybin and LSD received consistently high ratings for improving ED symptoms, overall mental health, and tolerability across all diagnostic groups. Preliminary evidence supports the use of psilocybin for AN, with several clinical trials under way.^20,24^ Trials of psychedelics for anxiety and depression have demonstrated lasting mental health benefits.^45,46,47^ The dataset supports further explorations of psychedelic-assisted psychotherapy for EDs including trials of LSD. Interestingly, MDMA and ketamine did not achieve the positive ratings evident with LSD and psilocybin, suggesting that classic psychedelics may possess unique properties in this regard.

The lifetime co-occurrence of EDs and substance use disorders is estimated to be as high as 50%,^16,17,48,49,50^ and tobacco, caffeine, and alcohol are highly represented. Accordingly, 49.3% of respondents in the current survey believed they had a problem with at least 1 substance, 15.2% of the cohort self-reported a comorbid drug dependence, and 9.3% reported alcohol use or dependence. While cannabis was strongly rated for beneficial effects on EDs and as a drug of choice, many daily cannabis users also reported problematic use.

### Limitations

This study has some limitations. First, our sample primarily involved high-income English-speaking countries and respondents with internet access. It may also have attracted a sample biased by having a strong interest in novelty-seeking and a history of positive experiences or attitudes toward drugs. There was a reliance on self-diagnosis without formal assessment of EDs or comorbid conditions. Recall bias may have affected self-reporting of drug use and clinical features. Some findings involve relatively small numbers of people with a specific diagnosis using a specific drug. Cannabis and psychedelics currently have a positive overall public perception, so ratings may have been influenced by expectation and placebo effects. As such, our results should be interpreted as exploratory rather than definitive.

## Conclusions

The findings of this survey study of prescription and nonprescription drug use among individuals with EDs highlight self-reported responses of the perceived benefits of psychedelics and cannabis for managing ED symptoms, often surpassing those of prescription medications. Nonetheless, prescription medications had positive ratings for general mental health. These findings suggest useful avenues for future clinical research to better validate the most beneficial pharmacotherapeutic approaches to treating different EDs.

## Notes

Supplement 1. **eAppendix.** MED-FED Survey **eTable 1.** Retention of Respondents Through the Sections of the Survey by Diagnosis **eTable 2.** Frequency of Distinct Diagnostic Categories **eTable 3.** Top Drugs of Choice Nominated by Respondents **eTable 4.** Most Problematic Drugs Nominated by Respondents

Supplement 2.
Data Sharing Statement
