# Supplementary material for: Prescription and Nonprescription Drug Use Among People With Eating Disorders
Source: JAMA Netw Open. 2025 Jul 22;8(7):e2522406. doi: 10.1001/jamanetworkopen.2025.22406 (PMC12284744; doi:10.1001/jamanetworkopen.2025.22406)
Supplement: Supplement 1. — eAppendix. MED-FED Survey eTable 1. Retention of Respondents Through the Sections of the Survey by Diagnosis eTable 2. Frequency of Distinct Diagnostic Categories eTable 3. Top Drugs of Choice Nominated by Respondents eTable 4. Most Problematic Drugs Nominated by Respondents [file jamanetwopen-e2522406-s001.pdf]

Supplemental Online Content

Rodan SC, Maguire S, Meez N, et al. Prescription and nonprescription drug use among people with eating disorders. *JAMA Netw Open*. 2025;8(7):e2522406. doi:10.1001/jamanetworkopen.2025.22406

eAppendix. MED-FED Survey
eTable 1. Retention of Respondents Through the Sections of the Survey by Diagnosis
eTable 2. Frequency of Distinct Diagnostic Categories
eTable 3. Top Drugs of Choice Nominated by Respondents
eTable 4. Most Problematic Drugs Nominated by Respondents

This supplemental material has been provided by the authors to give readers additional information about their work.

© 2025 Rodan SC et al. *JAMA Network Open*.

eAppendix. MED-FED Survey

© 2025 Rodan SC et al. *JAMA Network Open*.

Consent/eligibility page

Medication and other drugs for Eating Disorders - The MED-FED survey Welcome to the Medication and other drugs for Eating Disorders (MED-FED) online survey

The aim of this survey is to help us to better understand the lived experience of individuals with eating disorders. We will ask you about your medication and/or use of other drugs. The survey also explores personal experiences with these substances and the effects they have on managing your eating disorder and overall mental wellbeing.

This survey asks about:

1. You: Your age, gender, education, living and work situations

2. Your eating disorder diagnosis, symptoms and treatment

3. Your use of prescribed medications

4. Your use of caffeine

5. Your use of alcohol

6. Your use of tobacco and e-cigarettes

7. Your use of cannabis

8. Your use of stimulants

9. Your use of psychedelics

10. Your use of pro-social/party drugs (e.g. MDMA, ketamine, GHB)

11. Your use of opioids

12. Your use of any other drugs

Please read the Participant Information Statement before proceeding.

This survey should take around 10-20 minutes and is anonymous and confidential. Please read each question carefully and select responses that best reflect your experience.

Thank you for taking the time to participate in this research.

---

1) Eligibility

I confirm that I am:

18 or over Have a current eating disorder/
disordered eating that is causing significant distress
Confident in English

◯ Yes
◯ No

Participant Information Sheet

Please read the Participant Information Sheet attached

[Attachment: "Participant Information Sheet.pdf"]

Prof Iain McGregor
Director of Lambert Initiative for Cannabinoid Therapeutics
Dr Sarah Maguire
Director of InsideOut Institute for Eating Disorders
Ms Sarah-Catherine Rodan
Chief Investigator
Ms Kayla Greenstien
Co-investigator

Lambert Initiative for Cannabinoid Therapeutics
Brain and Mind Centre, NSW 2050
InsideOut Institute for Eating Disorders
Charles Perkins Centre, NSW 2006
Camperdown, University of Sydney
Australia
sarah-catherine.rodan@sydney.edu.au

Medication and other drugs for Eating Disorders – The MED-FED Survey

PARTICIPANT INFORMATION SHEET

We invite you to take part in a research study that explores medication and other drugs used by individuals with an eating disorder.

- Before you decide to participate, it is important for you to understand why we are conducting this research and what it involves.
- Please take the time to carefully read this information sheet and discuss it with others if you wish.
- You are free to choose whether to take part in this survey. If you choose to not take part, it will not negatively affect you or your relationship with anyone at the University of Sydney.
- Ask us if anything is unclear or if you would like further information.

About this study

- There are few evidence-based drug treatments for eating disorders
- We are collecting information on what types of medications and other drugs people with disordered eating use
- We want to understand how these medications and other drugs are harmful or helpful for people with an eating disorder
- This research is being conducted in Australia but is open for completion by an international audience.

2) Informed Consent

In giving my consent I confirm:

I read the Participant Information Statement and understand the nature of my involvement in this study. I understand I can withdraw from the study at any time by exiting the browser. I understand that I can save my responses and return to complete this study at a later time by recording the User ID code provided when I select the "save and return" option at the end of any page.

○ I consent
○ I do not consent

NOTE: YOU CAN EDIT YOUR RESPONSES BY CLICKING ON THE SURVEY QUEUE ICON ON THE TOP RIGHT CORNER OF THIS PAGE.

Instructions

Instructions:

- This survey is entirely anonymous and confidential. You have been allocated a user ID code that prevents responses from being matched to any personal and/or identifying information.

- You will be asked to define your use of illicit drugs as recreational or medicinal:

Recreational use is the use of a drug to induce an altered state of mind either for pleasure or other non-medical reason. Medicinal use is the use of a drug to treat, lessen or prevent unwanted/undesirable physical or mental health symptoms. - If you want to edit your previous responses you can do this by clicking on the SURVEY QUEUE icon on the top right corner of this page.

Thanks again for taking the time to complete this survey!

Section 1: Demographics

2% Complete

SECTION 1: YOU

Please provide some basic information about yourself.

How old are you?

____

What is your biological sex?

- ◯ Male
- ◯ Female
- ◯ Intersex
- ◯ Prefer not to say

Which of the following best describes your gender?

- ◯ Man
- ◯ Woman
- ◯ Non-binary / gender fluid
- ◯ Different Identity
- ◯ Prefer not to say

What is your country of residence?

◯ Afghanistan
◯ Albania
◯ Algeria
◯ Andorra
◯ Angola
◯ Antigua & Deps
◯ Argentina
◯ Armenia
◯ Australia
◯ Austria
◯ Azerbaijan
◯ Bahamas
◯ Bahrain
◯ Bangladesh
◯ Barbados
◯ Belarus
◯ Belgium
◯ Belize
◯ Benin
◯ Bhutan
◯ Bolivia
◯ Bosnia Herzegovina
◯ Botswana
◯ Brazil
◯ Brunei
◯ Bulgaria
◯ Burkina
◯ Burundi
◯ Cambodia
◯ Cameroon
◯ Canada
◯ Cape Verde
◯ Central African Rep
◯ Chad
◯ Chile
◯ China
◯ Colombia
◯ Comoros
◯ Congo
◯ Congo {Democratic Rep}
◯ Costa Rica
◯ Croatia
◯ Cuba
◯ Cyprus
◯ Czech Republic
◯ Denmark
◯ Djibouti
◯ Dominica
◯ Dominican Republic
◯ East Timor
◯ Ecuador
◯ Egypt
◯ El Salvador
◯ Equatorial Guinea
◯ Eritrea
◯ Estonia
◯ Ethiopia
◯ Fiji
◯ Finland
◯ France
◯ Gabon
◯ Gambia
◯ Georgia
◯ Germany
◯ Ghana
◯ Greece
◯ Grenada
◯ Guatemala
◯ Guinea

◯ Guinea-Bissau
◯ Guyana
◯ Haiti
◯ Honduras
◯ Hungary
◯ Iceland
◯ India
◯ Indonesia
◯ Iran
◯ Iraq
◯ Ireland {Republic}
◯ Israel
◯ Italy
◯ Ivory Coast
◯ Jamaica
◯ Japan
◯ Jordan
◯ Kazakhstan
◯ Kenya
◯ Kiribati
◯ Korea North
◯ Korea South
◯ Kosovo
◯ Kuwait
◯ Kyrgyzstan
◯ Laos
◯ Latvia
◯ Lebanon
◯ Lesotho
◯ Liberia
◯ Libya
◯ Liechtenstein
◯ Lithuania
◯ Luxembourg
◯ Macedonia
◯ Madagascar
◯ Malawi
◯ Malaysia
◯ Maldives
◯ Mali
◯ Malta
◯ Marshall Islands
◯ Mauritania
◯ Mauritius
◯ Mexico
◯ Micronesia
◯ Moldova
◯ Monaco
◯ Mongolia
◯ Montenegro
◯ Morocco
◯ Mozambique
◯ Myanmar, {Burma}
◯ Namibia
◯ Nauru
◯ Nepal
◯ Netherlands
◯ New Zealand
◯ Nicaragua
◯ Niger
◯ Nigeria
◯ Norway
◯ Oman
◯ Pakistan
◯ Palau
◯ Palestine
◯ Panama
◯ Papua New Guinea
◯ Paraguay
◯ Peru
◯ Philippines

◯ Poland
◯ Portugal
◯ Qatar
◯ Romania
◯ Russian Federation
◯ Rwanda
◯ St Kitts & Nevis
◯ St Lucia
◯ Saint Vincent & the Grenadines
◯ Samoa
◯ San Marino
◯ Sao Tome & Principe
◯ Saudi Arabia
◯ Senegal
◯ Serbia
◯ Seychelles
◯ Sierra Leone
◯ Singapore
◯ Slovakia
◯ Slovenia
◯ Solomon Islands
◯ Somalia
◯ South Africa
◯ South Sudan
◯ Spain
◯ Sri Lanka
◯ Sudan
◯ Suriname
◯ Swaziland
◯ Sweden
◯ Switzerland
◯ Syria
◯ Taiwan
◯ Tajikistan
◯ Tanzania
◯ Thailand
◯ Togo
◯ Tonga
◯ Trinidad & Tobago
◯ Tunisia
◯ Turkey
◯ Turkmenistan
◯ Tuvalu
◯ Uganda
◯ Ukraine
◯ United Arab Emirates
◯ United Kingdom
◯ United States
◯ Uruguay
◯ Uzbekistan
◯ Vanuatu
◯ Vatican City
◯ Venezuela
◯ Vietnam
◯ Yemen
◯ Zambia
◯ Zimbabwe

What is your ethnicity?

◯ White
◯ Hispanic or Latino
◯ Black or African American
◯ Native American or American Indian
◯ Asian
◯ Pacific Islander
◯ Aboriginal
◯ Torres Strait Islander
◯ Other
◯ Prefer not to say

____

What is your highest level of education?

◯ Elementary/Primary School
◯ Secondary/ High School
◯ Trade or Vocational training (e.g. Technical and Further Education (TAFE) or college)
◯ Undergraduate University degree (e.g. Bachelors, Honours)
◯ Postgraduate University degree (e.g. Masters, PhD)
◯ Other

Which best describes your current employment status?

◯ Full-time work
◯ Part-time work
◯ Home duties
◯ Unemployed
◯ Retired
◯ Disability pension
◯ Student
◯ Other

Which of the following best describes the sector you have been employed in for the majority of your working life?

☐ Agriculture, Forestry, Fishing and Hunting
☐ Computer, Software and Electronics Manufacturing
☐ Arts, Entertainment, and Recreation
☐ Finance
☐ Religious
☐ Legal Services
☐ Primary/Secondary Education
☐ College, University and Adult Education
☐ Military
☐ Hospitality
☐ Retail
☐ Office
☐ Healthcare
☐ Public service (e.g. Government)
☐ Law enforcement
☐ Education/ Academia
☐ Scientist
☐ Broadcasting and Telecommunications
☐ Athlete
☐ Manufacturing
☐ Construction
☐ Transport
☐ Mining
☐ Never employed
☐ Other

Please specify other

____

Where did you first hear about this survey?

◯ Facebook
◯ Instagram
◯ Twitter
◯ Snapchat
◯ Tiktok
◯ Other social media
◯ Reddit
◯ Other online forum (e.g. Whirlpool, Bluelight)
◯ Peak body communication services (e.g. Butterfly, BEAT, InsideOut, Lambert Initiative)
◯ Mailout
◯ A friend
◯ Doctor/healthcare provider
◯ Clinician/therapist/psychiatrist
◯ Hospital
◯ Private clinic
◯ Media (newspaper, radio, TV)
◯ SurveyCircle
◯ Other

Did you hear about this survey through any other sources?

____

Section 2: Details on ED

11% Complete

SECTION 2

The following questions will ask about your eating disorder diagnosis, symptoms and treatment.

Question	Response
Have you ever received a clinical diagnosis for your eating disorder?	◯ Yes<br>◯ No
Please select all diagnoses that apply to you.	☐ Anorexia Nervosa (AN)<br>☐ Bulimia Nervosa (BN)<br>☐ Binge Eating Disorder (BED)<br>☐ Other Specified Feeding or Eating Disorder (OSFED)<br>☐ Unspecified Feeding or Eating Disorder (UFED)<br>☐ Avoidant/Restrictive Food Intake Disorder (ARFID)<br>☐ Rumination Disorder<br>☐ Pica
Please select all disordered eating habits that apply to you.	☐ Stringent rules (e.g. calorie restriction) to influence shape or weight<br>☐ Long periods of time (8 waking hours or more) without eating<br>☐ Fear of food, eating and weight gain<br>☐ Self-induced vomiting<br>☐ Misuse of laxatives, diuretics or enemas<br>☐ Excessive exercise<br>☐ Repeated regurgitation of food<br>☐ Regular binge eating (eat unusually large amounts of food/loss of control over eating)<br>☐ Persistent eating of nonnutritive and nonfood substances<br>☐ Lack of interest in eating or food<br>☐ Avoidance of eating based on the sensory characteristics of food
At what age did you first recognise you may have an eating disorder?	______________________
Have you ever had a period of recovery followed by relapse?	◯ Yes<br>◯ No
How many times have you experienced a relapse of your eating disorder?	◯ 1<br>◯ 2<br>◯ 3<br>◯ 4<br>◯ 5+

Do you currently identify as having any of the following mental health conditions? Please select all that apply.	☐ Post-Traumatic Stress Disorder (PTSD)<br>☐ Complex PTSD<br>☐ Depression (of any type)<br>☐ Bipolar Disorder (of any type)<br>☐ Schizophrenia (of any type)<br>☐ Autism Spectrum Disorder (ASD)<br>☐ Obsessive Compulsive Disorder (OCD)<br>☐ Social Anxiety Disorder<br>☐ Generalised Anxiety Disorder (GAD)<br>☐ Panic Disorder<br>☐ Alcohol abuse or dependence<br>☐ Drug abuse or dependence<br>☐ Body Dysmorphic disorder<br>☐ Borderline Personality Disorder (BPD)<br>☐ Dissociative Identify Disorder (DID)<br>☐ Attention Deficit Hyperactivity Disorder (ADHD) (of any type)<br>☐ Other not mentioned<br>☐ Not applicable
Please specify any other mental health conditions you currently experience.	__________________________
Have you experienced any of the following mental health conditions in the past but now identify as recovered or in remission? Please select all that apply.	☐ Post-traumatic stress disorder (PTSD)<br>☐ Complex PTSD<br>☐ Depression (of any type)<br>☐ Bipolar disorder (of any type)<br>☐ Schizophrenia (of any type)<br>☐ Obsessive compulsive disorder (OCD)<br>☐ Social anxiety disorder<br>☐ Generalised anxiety disorder<br>☐ Panic disorder<br>☐ Alcohol abuse or dependence<br>☐ Drug abuse or dependence<br>☐ Body dysmorphic disorder<br>☐ Borderline personality disorder (BPD)<br>☐ Dissociative Identity disorder (DID)<br>☐ Attention deficit hyperactivity disorder (ADHD) (of any type)<br>☐ Other not mentioned<br>☐ Not applicable
Have you experience any other mental health conditions?	__________________________
Have you ever been hospitalised for an eating disorder?	○ Yes<br>○ No
How many times have you been hospitalised for an eating disorder?	○ 1<br>○ 2<br>○ 3<br>○ 4<br>○ 5<br>○ 6<br>○ 7<br>○ 8<br>○ 9<br>○ 10+

Have you ever engaged in psychological therapy? If yes, please select all applicable.	☐ Cognitive Behavioural Therapy (CBT)<br>☐ Family Based Treatment (FBT)/ Maudsley Model (MANTRA)<br>☐ Supportive Specialist Clinical Management (SSCM)<br>☐ Medical Nutrition Therapy<br>☐ Schema Therapy<br>☐ Dialectal Behavioural Therapy (DBT)<br>☐ Acceptance and Commitment Therapy (ACT)<br>☐ Art Therapy<br>☐ Dance Movement Therapy<br>☐ Equine Therapy<br>☐ Exposure and Response Prevention Therapy (ERP)<br>☐ Interpersonal Psychotherapy (IPT)<br>☐ Psychodynamic therapy<br>☐ Individual Therapy<br>☐ Other (please specify)<br>☐ Unsure which type<br>☐ Not applicable (never done therapy)
Please specify any other type psychotherapy you have received.	__________________________________

Throughout this survey we will ask you about how medication and other drugs effect your eating disorder symptoms. By this we mean symptoms such as, but are not limited to:

restrict your food intake restrict your calorie intake restrict whole food groups excessive exercise purging laxative abuse excessive body checking bingeing/ loss of control of eating

NOTE: YOU CAN EDIT YOUR PREVIOUS RESPONSES BY CLICKING ON THE SURVEY QUEUE ICON ON THE TOP RIGHT CORNER OF THIS PAGE.

Section 3: Prescribed medications

20% Complete

SECTION 3

The following questions ask about your history and current use of psychiatric medication ie. any medication prescribed by a doctor to help with your eating disorder, mood, attention, anxiety, sleep, obsessive thoughts or delusions.

Have you ever been prescribed a psychiatric medication?	◯ Yes<br>◯ No
Are you currently taking any psychiatric medication?	◯ Yes<br>◯ No
How many different types of prescribed psychiatric medications are you currently taking?	◯ 1<br>◯ 2<br>◯ 3<br>◯ 4<br>◯ 5 or more

Please have your prescribed psychiatric medications in front of you to answer the following questions.

Name of medication ______
How often do you take this medication? ______
This medication makes my eating disorder symptoms better. ______
This medication makes my eating disorder symptoms worse. ______
This medication has overall benefits for my mental health. ______
This medication has unpleasant side effects. ______
Any other comments that you would like to share about this medication? (optional) ______

Please have your psychiatric medications in front of you to answer the following questions.

Psychiatric drug 1

Name of medication ______
How often do you take this medication? ______
This medication makes my eating disorder symptoms better. ______
This medication makes my eating disorder symptoms worse. ______
This medication has overall benefits for my mental health. ______
This medication has unpleasant side effects. ______
Any other comments that you would like to share about this medication? (optional) ______

Psychiatric drug 2

Name of medication ______
How often do you take this medication? ______
This medication makes my eating disorder symptoms better. ______
This medication makes my eating disorder symptoms worse. ______
This medication has overall benefits for my mental health. ______
This medication has unpleasant side effects. ______
Any other comments that you would like to share about this medication? (optional) ______

Please have your psychiatric medications in front of you to answer the following questions.

Psychiatric drug 1

Name of medication ______
How often do you take this medication? ______
This medication makes my eating disorder symptoms better. ______
This medication makes my eating disorder symptoms worse. ______
This medication has overall benefits for my mental health. ______
This medication has unpleasant side effects. ______
Any other comments that you would like to share about this medication? (optional) ______

Psychiatric drug 2

Name of medication ______
How often do you take this medication? ______
This medication makes my eating disorder symptoms better. ______
This medication makes my eating disorder symptoms worse. ______
This medication has overall benefits for my mental health. ______
This medication has unpleasant side effects. ______
Any other comments that you would like to share about this medication? (optional) ______

Psychiatric drug 3

Name of medication ______
How often do you take this medication? ______
This medication makes my eating disorder symptoms better. ______
This medication makes my eating disorder symptoms worse. ______
This medication has overall benefits for my mental health. ______
This medication has unpleasant side effects. ______
Any other comments that you would like to share about this medication? (optional) ______

Please have your psychiatric medications in front of you to answer the following questions.

Psychiatric drug 1

Name of medication ______
How often do you take this medication? ______
This medication makes my eating disorder symptoms better. ______
This medication makes my eating disorder symptoms worse. ______
This medication has overall benefits for my mental health. ______
This medication has unpleasant side effects. ______
Any other comments that you would like to share about this medication? (optional) ______

Psychiatric drug 2

Name of medication ______
How often do you take this medication? ______
This medication makes my eating disorder symptoms better. ______
This medication makes my eating disorder symptoms worse. ______
This medication has overall benefits for my mental health. ______
This medication has unpleasant side effects. ______
Any other comments that you would like to share about this medication? (optional) ______

Psychiatric drug 3

Name of medication ______
How often do you take this medication? ______
This medication makes my eating disorder symptoms better. ______
This medication makes my eating disorder symptoms worse. ______
This medication has overall benefits for my mental health. ______
This medication has unpleasant side effects. ______
Any other comments that you would like to share about this medication? (optional) ______

Psychiatric drug 4

Name of medication ______
How often do you take this medication? ______
This medication makes my eating disorder symptoms better. ______
This medication makes my eating disorder symptoms worse. ______
This medication has overall benefits for my mental health. ______
This medication has unpleasant side effects. ______
Any other comments that you would like to share about this medication? (optional) ______

Please have your psychiatric medications in front of you to answer the following questions and answer the following questions for up to 5 of your psychiatric drugs.

Psychiatric drug 1

Name of medication ______
How often do you take this medication? ______
This medication makes my eating disorder symptoms better. ______
This medication makes my eating disorder symptoms worse. ______
This medication has overall benefits for my mental health. ______
This medication has unpleasant side effects. ______
Any other comments that you would like to share about this medication? (optional) ______

Psychiatric drug 2

Name of medication ______
How often do you take this medication? ______
This medication makes my eating disorder symptoms better. ______
This medication makes my eating disorder symptoms worse. ______
This medication has overall benefits for my mental health. ______
This medication has unpleasant side effects. ______
Any other comments that you would like to share about this medication? (optional) ______

Psychiatric drug 3

Name of medication ______
How often do you take this medication? ______
This medication makes my eating disorder symptoms better. ______
This medication makes my eating disorder symptoms worse. ______
This medication has overall benefits for my mental health. ______
This medication has unpleasant side effects. ______
Any other comments that you would like to share about this medication? (optional) ______

Psychiatric drug 4

Name of medication ______
How often do you take this medication? ______
This medication makes my eating disorder symptoms better. ______
This medication makes my eating disorder symptoms worse. ______
This medication has overall benefits for my mental health. ______
This medication has unpleasant side effects. ______
Any other comments that you would like to share about this medication? (optional) ______

Psychiatric drug 5

Name of medication ______
How often do you take this medication? ______
This medication makes my eating disorder symptoms better. ______
This medication makes my eating disorder symptoms worse. ______
This medication has overall benefits for my mental health. ______
This medication has unpleasant side effects. ______
Any other comments that you would like to share about this medication? (optional) ______

Apart from your current medications, how many other psychiatric medications have you been prescribed in your lifetime?

◯ 0
◯ 1
◯ 2
◯ 3
◯ 4
◯ 5
◯ 6
◯ 7
◯ 8
◯ 9
◯ 10+
◯ 15+

If you are able to recall, please name any of the other psychiatric medications you have been prescribed (optional). ______________________________

NOTE: YOU CAN EDIT YOUR PREVIOUS RESPONSES BY CLICKING ON THE SURVEY QUEUE ICON ON THE TOP RIGHT CORNER OF THIS PAGE..

Section 4: Caffeine

29% Complete

SECTION 4: CAFFEINE

The following questions ask about your consumption of caffeine either through beverages such as coffee and tea, or other forms of caffeine such as tablets.

Have you ever consumed caffeine (this includes tea, coffee, soda, tablets etc.) ?	◯ Yes<br>◯ No
Have you consumed caffeine in the past 12 months?	◯ Yes<br>◯ No

Caffeine

How do you consume caffeine? (please select all that apply to you) ______

How often do you have caffeine? ______

How many units of caffeine do you have on a typical day when you consume caffeine (consider one unit as one drink, pill or powder serving)? ______

I need to have caffeine every day ______

I would get a headache if I went without caffeine ______

Caffeine allows me to skip meals ______

Caffeine suppresses feelings of hunger. ______

How would you describe your use of caffeine? ______

Caffeine makes my eating disorder symptoms better. ______

Caffeine makes my eating disorder symptoms worse. ______

Caffeine has overall benefits for my mental health. ______

Caffeine has unpleasant side effects. ______

Do you believe you have a problem with caffeine? ______

Any other comments that you would like to share about your use of caffeine? (optional) ______

Which caffeinated beverage do you most commonly consume? (optional)	◯ Long black/Americano<br>◯ Espresso<br>◯ Flat white<br>◯ Cappucino<br>◯ Latte<br>◯ Instant coffee<br>◯ Green tea<br>◯ Black tea<br>◯ Black tea + milk<br>◯ Diet Coke<br>◯ Coke<br>◯ Pepsi<br>◯ Red bull<br>◯ Monster Energy<br>◯ Other
Please specify other caffeinated beverage	______________________________

NOTE: YOU CAN EDIT YOUR PREVIOUS RESPONSES BY CLICKING ON THE SURVEY QUEUE ICON ON THE TOP RIGHT CORNER OF THIS PAGE.

Section 5. Alcohol

29% Complete

SECTION 5: ALCOHOL

This section will ask about your use of alcohol.

Have you consumed any alcohol in the past 12 months?
◯ Yes
◯ No

Alcohol

How often do you drink alcohol? ______
How many drinks containing alcohol do you have on a typical day when you drink? ______
How often do you have six or more drinks on one occasion? ______
How would you describe your use of alcohol? ______
Alcohol makes eating disorder symptoms better. ______
Alcohol makes my eating disorder symptoms worse. ______
Alcohol has overall benefits for my mental health. ______
Alcohol has unpleasant side effects. ______
Do you believe you have a problem with alcohol? ______
Any other comments you would like to share about your use of alcohol? (optional) ______

NOTE: YOU CAN EDIT YOUR PREVIOUS RESPONSES BY CLICKING ON THE SURVEY QUEUE ICON ON THE TOP RIGHT CORNER OF THIS PAGE.

Section 6: Tobacco, Nicotine

38% Complete

SECTION 6: TOBACCO & NICOTINE

This section will ask about your use of tobacco and nicotine.

Have you smoked tobacco in the past 12 months? ◯ Yes ◯ No

Tobacco

How often do you smoke cigarettes? ______
How would you describe your use of tobacco? ______
Tobacco makes my eating disorder symptoms better. ______
Tobacco makes my eating disorder symptoms worse. ______
Tobacco has overall benefits for my mental health. ______
Tobacco has unpleasant side effects. ______
Do you believe you have a problem with tobacco? ______
Any other comments you would like to share about your use of tobacco? (optional) ______

How soon after you wake up do you smoke your first cigarette? ______
Do you find it difficult to refrain from smoking in places where it is forbidden (e.g. in church, at the library, in the cinema)? ______
Which cigarette would you hate to give up the most? ______
How many cigarettes per day do you smoke? ______
Do you smoke more frequently during the first hours after waking than during the rest of the day? ______
Do you smoke when you are so ill that you are in bed most of the day? ______

Have you vaped nicotine in the past 12 months? ◯ Yes ◯ No

Nicotine

How often do you vape nicotine? ______
How would you describe your use of nicotine vaping? ______
Vaping nicotine makes my eating disorder symptoms better. ______
Vaping nicotine makes my eating disorder symptoms worse. ______
Vaping nicotine has overall benefits for my mental health. ______
Vaping nicotine has unpleasant side effects. ______
Do you believe you have a problem with vaping nicotine ? ______
Any other comments you would like to share about your use of nicotine? (optional) ______

How many times per day do you usually use your nicotine vape? (assume that one "time" consists of around 15 puffs or lasts around 10 minutes)

______

Do you find it difficult to refrain from vaping in places where it is forbidden (e.g. in church, at the library, in the cinema)?

______

When would you hate most to give up nicotine vape use?

______

On days that you can use your nicotine vape freely, how soon after you wake up do you first use your vape?

______

Do you use your nicotine vape more frequently during the first two hours of the day than during the rest of the day?

______

Do you use your nicotine vape when you are so ill that you are in bed most of the day?

______

NOTE: YOU CAN EDIT YOUR PREVIOUS RESPONSES BY CLICKING ON THE SURVEY QUEUE ICON ON THE TOP RIGHT CORNER OF THIS PAGE.

Section 7: Cannabis

47% Complete

SECTION 7: CANNABIS

The following questions will ask about your use of naturally derived cannabis products (e.g weed, bud, flower, CBD oil, capsules, edibles, dabs, shatter, etc).

We will also ask if you have ever used synthetic cannabis (e.g. K2, spice).

The legality of cannabis varies across countries. We will ask you to specify whether you have sourced your cannabis:

With a prescription from a doctor Legally without a prescription (e.g. sourced at a dispensary, over the counter, health store, online) Illegally

Have you ever used cannabis, synthetic cannabis or cannabis-related products?	◯ Yes<br>◯ No
Have you used cannabis, synthetic cannabis or cannabis-related products in the past 12 months?	◯ Yes<br>◯ No
What types of cannabis products do you use?	☐ Bud/flower/weed (smoked or vaped)<br>☐ Oral edibles (e.g. cookies, gummies etc.)<br>☐ Dabs, shatter, wax<br>☐ Vape liquid<br>☐ Cannabis oil (e.g. liquid extract cannabis/CBD oils or tincture)<br>☐ Wafer<br>☐ Tablets/capsules<br>☐ Oral spray (mouth or oromucosal)<br>☐ Synthetic cannabis (e.g. spice, K2)<br>☐ Other (please specify)

Cannabis bud/flower/weed (smoked or vaped)

How would you describe the content of your cannabis bud/flower? ______

______
How do you source your cannabis bud/flower? ______
How would you describe your use of cannabis bud/flower? ______
How often do you use cannabis bud/flower? ______
Cannabis bud/flower makes my eating disorder symptoms better. ______
Cannabis bud/flower makes my eating disorder symptoms worse. ______
Cannabis bud/flower has overall benefits for my mental health. ______
Cannabis bud/flower has unpleasant side effects. ______
Do you believe you have a problem with cannabis bud/flower? ______
Any other comments that you would like to share about your use of cannabis bud/flower? (optional) ______

Cannabis edibles

How would you describe the content of your cannabis edibles. ______

______
How do you source your cannabis edibles? ______
How often do you use cannabis edibles? ______
Please describe your use of cannabis edibles. ______

Cannabis edibles make my eating disorder symptoms better. ______
Cannabis edibles make my eating disorder symptoms worse. ______
Cannabis edibles has overall benefits for my mental health. ______
Cannabis edibles has unpleasant side effects. ______
Do you believe you have a problem with cannabis edibles? ______
Any other comments that you would like to share about your use of cannabis edibles? (optional) ______

Cannabis dabs/shatter/wax

How would you describe the content of your cannabis dabs. ______

______

How do you source your cannabis dabs? ______
How would you describe your use of cannabis dabs? ______
How often do you use cannabis dabs? ______
Cannabis dabs make my eating disorder symptoms better. ______
Cannabis dabs make my eating disorder symptoms worse. ______
Cannabis dabs has overall benefits for my mental health. ______
Cannabis dabs has unpleasant side effects. ______
Do you believe you have a problem with cannabis dabs? ______
Any other comments that you would like to share about your use of cannabis dabs? (optional) ______

Cannabis liquid (vaped)

How would you describe the content of your cannabis vape liquid. ______

______

How do you source your cannabis vape liquid? ______
How would you describe your use of cannabis vape liquid? ______
How often do you use cannabis vape liquid? ______
Cannabis vape liquid makes my eating disorder symptoms better. ______
Cannabis vape liquid makes my eating disorder symptoms worse. ______
Cannabis vape liquid has overall benefits for my mental health. ______
Cannabis vape liquid has unpleasant side effects. ______
Do you believe you have a problem with cannabis vape liquid? ______
Any other comments that you would like to share about your use of cannabis vape liquid? (optional) ______

Cannabis oil (liquid extract cannabis)

How would you describe the content of your cannabis oil. ______
How do you source your cannabis oil. ______
How would you describe your use of cannabis oil? ______
How often do you use cannabis oil? ______
Cannabis oil makes my eating disorder symptoms better. ______
Cannabis oil makes my eating disorder symptoms worse. ______
Cannabis oil has overall benefits for my mental health. ______
Cannabis oil has unpleasant side effects. ______
Do you believe you have a problem with cannabis oil? ______
Any other comments that you would like to share about your use of cannabis oil? (optional) ______

Please specify.

____

Wafer

How would you describe the content of your wafers. ______

______

How do you source your wafers. ______
Describe your use of wafers. ______
How often do you use wafers? ______
Wafers makes my eating disorder symptoms better. ______
Wafers makes my eating disorder symptoms worse. ______
Wafers has overall benefits for my mental health. ______
Wafers has unpleasant side effects. ______
Do you believe you have a problem with wafers? ______
Any other comments that you would like to share about your use of wafers? (optional) ______

Capsules/tablets

How would you best describe the content of your cannabis capsules? ______

______

How do you source your capsules? ______
How often are you taking capsules? ______
Please describe your use of capsules. ______
Capsules make my eating disorder symptoms better. ______
Capsules make my eating disorder symptoms worse. ______
Capsules has overall benefits for my mental health. ______
Capsules has unpleasant side effects. ______
Do you believe you have a problem with capsules? ______
Any other comments that you would like to share about your use of capsules? (optional) ______

Spray

How would you best describe the content of your cannabis spray? ______

______

How do you source your cannabis spray? ______
How often do you use cannabis spray? ______
Please describe your use of cannabis spray? ______
Cannabis spray makes my eating disorder symptoms better. ______
Cannabis spray makes my eating disorder symptoms worse. ______
Cannabis spray has overall benefits for my mental health. ______
Cannabis spray has unpleasant side effects. ______
Do you believe you have a problem with cannabis spray? ______
Any other comments that you would like to share about your use of cannabis spray ? (optional) ______

Other cannabis

What other cannabis products do you use? ______
How would you best describe the content of your cannabis? ______

______

How do you source this cannabis? ______
How often do you use this cannabis? ______
Please describe your use of this cannabis spray? ______
This cannabis product makes my eating disorder symptoms better. ______
This cannabis product makes my eating disorder symptoms worse. ______
This cannabis product has overall benefits for my mental health. ______
This cannabis product has unpleasant side effects. ______
Do you believe you have a problem with this cannabis product ? ______
Any other comments that you would like to share about your use of this cannabis product ? (optional) ______

Synthetic Cannabis (eg. spice, K2)

How would you best describe the content of your synthetic cannabis? ______

______

How often do you use synthetic cannabis? ______
How would you describe your use of synthetic cannabis? ______
Synthetic cannabis makes my eating disorder symptoms better. ______
Synthetic cannabis makes my eating disorder symptoms worse. ______
Synthetic cannabis has overall benefits for my mental health. ______
Synthetic cannabis has unpleasant side effects. ______
Do you believe you have a problem with spice ? ______
Any other comments that you would like to share about your use of spice ? (optional) ______

NOTE: YOU CAN EDIT YOUR PREVIOUS RESPONSES BY CLICKING ON THE SURVEY QUEUE ICON ON THE TOP RIGHT CORNER OF THIS PAGE.

Section 8: Stimulants

55% Complete

SECTION 8: STIMULANTS

The following questions will ask about your use of illegally sourced stimulants (e.g. cocaine, meth, speed, prescription stimulants).

We will also ask you to specify the routes of administration that you use.

Have you ever used illegally sourced stimulants?	◯ Yes<br>◯ No
Have you used an illegally sourced stimulant in the past 12 months?	◯ Yes<br>◯ No
Please select all the illegally sourced stimulants and amphetamines that you have used in the past 12 months.	☐ Cocaine (e.g. coke, gear, blow)<br>☐ Methamphetamine (e.g. crystal meth/Ice)<br>☐ Amphetamines (e.g. speed)<br>☐ Mephedrone (e.g. MCAT, meow meow)<br>☐ Other Bath Salts (e.g. flakka)<br>☐ Illegally sourced prescription stimulants (e.g. Ritalin, Adderall, Dexedrine, Vyvanse)<br>☐ Other (please specify)

Cocaine

How often do you use cocaine? ______
How do you consume cocaine? ______
How would you describe your use of cocaine? ______
Cocaine makes my eating disorder symptoms better. ______
Cocaine makes my eating disorder symptoms worse. ______
Cocaine has overall benefits for my mental health. ______
Cocaine has unpleasant side effects. ______
Do you believe you have a problem with cocaine? ______
Any other comments that you would like to share about your use of cocaine? (optional) ______

Methamphetamine (Crystal Meth)

How often do you use methamphetamine? ______
How do you consume methamphetamine? ______
How would you describe your use of methamphetamine? ______
Methamphetamine makes my eating disorder symptoms better. ______
Methamphetamine makes my eating disorder symptoms worse. ______
Methamphetamine has overall benefits for my mental health. ______
Methamphetamine has unpleasant side effects. ______
Do you believe you have a problem with methamphetamine? ______
Any other comments that you would like to share about your use of methamphetamine? (optional) ______

Amphetamines (Speed)

How often do you use amphetamines? ______
How would you describe your use of amphetamines? ______
Amphetamines make my eating disorder symptoms better. ______
Amphetamines make my eating disorder symptoms worse. ______
Amphetamines has overall benefits for my mental health. ______
Amphetamines has unpleasant side effects. ______
Do you believe you have a problem with amphetamines? ______
Any other comments that you would like to share about your use of amphetamines? (optional) ______

Mephedrone (e.g. MCAT, Meow Meow)

How often do you use mephedrone? ______
How would you describe your use of mephedrone? ______
Mephedrone makes my eating disorder symptoms better. ______
Mephedrone makes my eating disorder symptoms worse. ______
Mephedrone has overall benefits for my mental health. ______
Mephedrone has unpleasant side effects. ______
Do you believe you have a problem with mephedrone? ______
Any other comments that you would like to share about your use of mephedrone? (optional) ______

Other Bath Salts

Name of bath salt. ______
How often do you use bath salts? ______
How would you describe your use of bath salts? ______
Bath salts make my eating disorder symptoms better. ______
Bath salts makes my eating disorse symptoms worse. ______
Bath salts has overall benefits for my mental health. ______
Bath salts has unpleasant side effects. ______
Do you believe you have a problem with bath salts? ______
Any other comments that you would like to share about your use of bath salts? (optional) ______

Other stimulant

Name of stimulant. ______
How often do you use this stimulant? ______
How would you describe your use of this stimulant? ______
This stimulant make my eating disorder symptoms better. ______
This stimulant makes my eating disorse symptoms worse. ______
This stimulant has overall benefits for my mental health. ______
This stimulant has unpleasant side effects. ______
Do you believe you have a problem with this stimulant? ______
Any other comments that you would like to share about your use of this stimulant? (optional) ______

How many different illegally sourced prescription stimulants have used in the past 12 months?

- ◯ 1
- ◯ 2
- ◯ 3+

Prescription stimulants 1

Name of stimulant. ______
How often do you use this stimulant? ______
How would you describe your use of stimulants? ______
This stimulant makes my eating disorder symptoms better. ______
This stimulant makes my eating disorder symptoms worse. ______
This stimulant has overall benefits for my mental health. ______
This stimulant has unpleasant side effects. ______
Do you believe you have a problem with this stimulant? ______
Any other comments that you would like to share about your use of this stimulant? (optional) ______

Prescription Stimulant 1

Name of stimulant. ______
How often do you use this stimulant? ______
How would you describe your use of stimulants? ______
This stimulant makes my eating disorder symptoms better. ______
This stimulant makes my eating disorder symptoms worse. ______
This stimulant has overall benefits for my mental health. ______
This stimulant has unpleasant side effects. ______
Do you believe you have a problem with this stimulant? ______
Any other comments that you would like to share about your use of this stimulant? (optional) ______
Prescription Stimulant 2

Name of stimulant. ______
How often do you use this stimulant? ______
How would you describe your use of stimulants? ______

This stimulant makes my eating disorder symptoms better. ______
This stimulant makes my eating disorder symptoms worse. ______
This stimulant has overall benefits for my mental health. ______
This stimulant has unpleasant side effects. ______
Do you believe you have a problem with this stimulant? ______
Any other comments that you would like to share about your use of this stimulant? (optional) ______

Prescription Stimulant 1

Name of stimulant. ______
How often do you take this stimulant? ______
How would you describe your use of stimulants? ______
This stimulant makes my eating disorder symptoms better. ______
This stimulant makes my eating disorder symptoms worse. ______
This stimulant has overall benefits for my mental health. ______
This stimulant has unpleasant side effects. ______
Do you believe you have a problem with this stimulant? ______
Any other comments that you would like to share about your use of this stimulant? (optional) ______

Prescription Stimulant 2

Name of stimulant. ______
How often do you use this stimulant? ______
How would you describe your use of stimulants? ______
This stimulant makes my eating disorder symptoms better. ______
This stimulant makes my eating disorder symptoms worse. ______
This stimulant has overall benefits for my mental health. ______
This stimulant has unpleasant side effects. ______
Do you believe you have a problem with this stimulant? ______
Any other comments that you would like to share about your use of this stimulant? (optional) ______

Prescription Stimulant 3

Name of stimulant. ______
How often do you take this stimulant? ______
How would you describe your use of stimulants? ______
This stimulant makes my eating disorder symptoms better. ______
This stimulant makes my eating disorder symptoms worse. ______
This stimulant has overall benefits for my mental health. ______
This stimulant has unpleasant side effects. ______
Do you believe you have a problem with this stimulant? ______
Any other comments that you would like to share about your use of this stimulant? (optional) ______

NOTE: YOU CAN EDIT YOUR PREVIOUS RESPONSES BY CLICKING ON THE SURVEY QUEUE ICON ON THE TOP RIGHT CORNER OF THIS PAGE.

Section 9: Psychedelics

64% Complete

SECTION 9: PSYCHEDELICS

The following questions will ask about your use of psychedelics e.g. LSD, magic mushrooms etc. This does NOT include drugs like MDMA and ketamine.

Have you ever used psychedelics (e.g. magic mushrooms, acid, DMT)?	◯ Yes<br>◯ No
Have you used psychedelic in the past 12 months?	◯ Yes<br>◯ No
Please select all of the psychedelics you have used in the past 12 months.	☐ Magic mushrooms (e.g. psilocybin)<br>☐ LSD (acid)<br>☐ Ayahuasca (South American brew containing DMT)<br>☐ DMT (e.g. N,N-DMT)<br>☐ 5-MeO-DMT (e.g. bufotenine/toad venom)<br>☐ Mescaline (e.g. San Pedro, Peruvian Torch, Peyote Cactus)<br>☐ Ibogaine (e.g. Iboga/African Shrub)<br>☐ Salvinorin A (e.g. Salvia)<br>☐ 2-CB (e.g tripstacy)<br>☐ Other (please specify)

Magic Mushrooms (e.g psilocybin, shrooms)

How often do you use magic mushrooms? ______
How would you describe your use of magic mushrooms? ______
Magic mushrooms make my eating disorder symptoms better. ______
Magic mushrooms makes my eating disorder symptoms worse. ______
Magic mushrooms has overall benefits for my mental health. ______
Magic mushrooms has unpleasant side effects. ______
Do you believe you have a problem with magic mushrooms? ______
Any other comments that you would like to share about your use of magic mushrooms? (optional) ______

LSD

How often do you use LSD? ______
How would you describe your use of LSD? ______
LSD makes my eating disorder symptoms better. ______
LSD makes my eating disorder symptoms worse. ______
LSD has overall benefits for my mental health. ______
LSD has unpleasant side effects. ______
Do you believe you have a problem with LSD? ______
Any other comments that you would like to share about your use LSD? (optional) ______

Ayahuasca

How often do you use ayahuasca? ______
How would you describe your use of ayahuasca? ______
Ayahuasca makes my eating disorder symptoms better. ______
Ayahuasca makes my eating disorder symptoms worse. ______
Ayahuasca has overall benefits for my mental health. ______
Ayahuasca has unpleasant side effects. ______
Do you believe you have a problem with ayahuasca? ______
Any other comments that you would like to share about your use of ayahuasca? (optional) ______

DMT

How often do you use DMT? ______
How would you describe your use of DMT? ______
DMT makes my eating disorder symptoms better. ______
DMT makes my eating disorder symptoms worse. ______
DMT has overall benefits for my mental health. ______
DMT has unpleasant side effects. ______
Do you believe you have a problem with DMT? ______
Any other comments that you would like to share about your use of DMT? (optional) ______

5-MeO-DMT

How often do you use 5-MeO-DMT? ______
How would you describe your use of 5-MeO-DMT? ______
5-MeO-DMT makes my eating disorder symptoms better. ______
5-MeO-DMT makes my eating disorder symptoms worse. ______
5-MeO-DMT has overall benefits for my mental health. ______
5-MeO-DMT has unpleasant side effects. ______
Do you believe you have a problem with 5-MeO-DMT? ______
Any other comments that you would like to share about your use of 5-MeO-DMT? (optional) ______

Mescaline

How often do you use mescaline? ______
How would you describe your use of mescaline? ______
Mescaline makes my eating disorder symptoms better. ______
Mescaline makes my eating disorder symptoms worse. ______
Mescaline has overall benefits for my mental health. ______
Mescaline has unpleasant side effects. ______
Do you believe you have a problem with mescaline? ______
Any other comments that you would like to share about your use of mescaline? (optional) ______

Ibogaine

How often do you use ibogaine? ______
How would you describe your use of ibogaine? ______
Ibogaine makes my eating disorder symptoms better. ______
Ibogaine makes my eating disorder symptoms worse. ______
Ibogaine has overall benefits for my mental health. ______
Ibogaine has unpleasant side effects. ______
Do you believe you have a problem with Ibogaine? ______
Any other comments that you would like to share about your use of Ibogaine? (optional) ______

Salvia

How often do you use salvia? ______
How would you describe your use of salvia? ______
Salvia makes my eating disorder symptoms better. ______
Salvia makes my eating disorder symptoms worse. ______
Salvia has overall benefits for my mental health. ______
Salvia has unpleasant side effects. ______
Do you believe you have a problem with salvia? ______
Any other comments that you would like to share about your use of salvia? (optional) ______

2-CB

How often do you use 2-CB? ______
How would you describe your use of 2-CB? ______
2-CB makes my eating disorder symptoms better. ______
2-CB makes my eating disorder symptoms worse. ______
2-CB has overall benefits for my mental health. ______
2-CB has unpleasant side effects. ______
Do you believe you have a problem with 2-CB? ______
Any other comments that you would like to share about your use of 2-CB? (optional) ______

Other psychedelic

Name of psychedelic. ______
How often do you use this psychedelic drug? ______
How would you describe your use of this psychedelic drug? ______
This psychedelic makes my eating disorder symptoms better. ______
This psychedelic makes my eating disorder symptoms worse. ______
This psychedelic has overall benefits for my mental health. ______
This psychedelic has unpleasant side effects. ______
Do you believe you have a problem with this psychedelic? ______
Any other comments that you would like to share about your use of this psychedelic? (optional) ______

NOTE: YOU CAN EDIT YOUR PREVIOUS RESPONSES BY CLICKING ON THE SURVEY QUEUE ICON ON THE TOP RIGHT CORNER OF THIS PAGE.

Section 10 Pro-Social/Party Drugs

73% Complete

SECTION 10: PRO-SOCIAL/PARTY DRUGS

The following questions will ask about your use of pro-social/party drugs e.g. MDMA, Ketamine, GHB, poppers, balloons, PCP.

We acknowledge that some of these drugs (ketamine and GHB) are available by prescription. If you are prescribed these medications for depression or narcolepsy, please include in Section 3: prescribed medications.

Have you ever used pro-social/party drugs?	◯ Yes<br>◯ No
Have you used any of the following substances in the past 12 months? Please select all that apply.	☐ MDMA (e.g. Ecstasy, Molly)<br>☐ Ketamine (e.g. special K)<br>☐ GHB (e.g. G, liquid ecstasy)<br>☐ PCP (e.g. angel dust)<br>☐ Nitrous Oxide (e.g. balloons/laughing gas)<br>☐ Poppers (e.g. jungle juice/amyl nitrate)<br>☐ No

MDMA

How often do you use MDMA? ______
How would you describe your use of MDMA? ______
MDMA makes my eating disorder symptoms better. ______
MDMA makes my eating disorder symptoms worse. ______
MDMA has overall benefits for my mental health. ______
MDMA has unpleasant side effects. ______
Do you believe you have a problem with MDMA? ______
Any other comments that you would like to share about your use of MDMA? (optional) ______

Ketamine

How often do you use Ketamine? ______
How would you describe your use of Ketamine? ______
Ketamine makes my eating disorder symptoms better. ______
Ketamine makes my eating disorder symptoms worse. ______
Ketamine has overall benefits for my mental health. ______
Ketamine has unpleasant side effects. ______
Do you believe you have a problem with ketamine? ______
Any other comments that you would like to share about your use of ketamine? (optional) ______

GHB

How often do you use GHB? ______
How would you describe your use of GHB? ______
GHB makes my eating disorder symptoms better. ______
GHB makes my eating disorder symptoms worse. ______
GHB has overall benefits for my mental health. ______
GHB has unpleasant side effects. ______
Do you believe you have a problem with GHB? ______
Any other comments that you would like to share about your use of GHB? (optional) ______

PCP

How often do you use PCP? ______
How would you describe your use of PCP? ______
PCP makes my eating disorder symptoms better. ______
PCP makes my eating disorder symptoms worse. ______
PCP has overall benefits for my mental health. ______
PCP has unpleasant side effects. ______
Do you believe you have a problem with PCP? ______
Any other comments that you would like to share about your use of PCP? (optional) ______

Nitrous Oxide (Balloons)

How often do you use nitrous oxide? ______
How would you describe your use of nitrous oxide? ______
Nitrous oxide makes my eating disorder symptoms better. ______
Nitrous oxide makes my eating disorder symptoms worse. ______
Nitrous oxide has overall benefits for my mental health. ______
Nitrous oxide has unpleasant side effects. ______
Do you believe you have a problem with nitrous oxide? ______
Any other comments that you would like to share about your use of nitrous oxide? (optional) ______

Poppers

How often do you use poppers? ______
How would you describe your use of poppers? ______
Poppers makes my eating disorder symptoms better. ______
Poppers makes my eating disorder symptoms worse. ______
Poppers has overall benefits for my mental health. ______
Poppers has unpleasant side effects. ______
Do you believe you have a problem with poppers? ______
Any other comments that you would like to share about your use of poppers? (optional) ______

NOTE: YOU CAN EDIT YOUR PREVIOUS RESPONSES BY CLICKING ON THE SURVEY QUEUE ICON ON THE TOP RIGHT CORNER OF THIS PAGE.

Section 11: Opioids

82% Complete

SECTION 11: OPIOIDS

The following questions will ask about your use of opioids. This refers to heroin, fentanyl and other prescription opioids (e.g. morphine, codeine, oxycodone).

We will ask you to specify whether you have sourced your opioids:

With a prescription from a doctor Legally without a prescription (e.g. over the counter) Illegally

Have you ever used opioids?

- ◯ Yes
- ◯ No

Have you used any of the following opioids in the past 12 months?

- ☐ Heroin
- ☐ Morphine
- ☐ Fentanyl
- ☐ Methadone
- ☐ Oxycodone (e.g. Oxycontin, Percocet)
- ☐ Hydrocodone (e.g. Vicodin)
- ☐ Hydromorphone (e.g. Dilaudid)
- ☐ Tapentadol (e.g. Palexia)
- ☐ Codeine
- ☐ Tramadol
- ☐ Other opioid (please specify)
- ☐ No

Heroin

How do you source your heroin? ______
How do you take your heroin? ______
How often do you use heroin? ______
How would you describe your use of heroin? ______
Heroin makes my eating disorder symptoms better. ______
Heroin makes my eating disorder symptoms worse. ______
Heroin has overall benefits for my mental health. ______
Heroin has unpleasant side effects. ______
Do you believe you have a problem with heroin? ______
Any other comments that you would like to share about your use of heroin? (optional) ______

Morphine

How do you source your morphine? ______
How often do you use morphine? ______
How would you describe your use of morphine? ______
Morphine makes my eating disorder symtoms better. ______
Morphine makes my eating disorder symptoms worse. ______
Morphine has overall benefits for my mental health. ______
Morphine has unpleasant side effects. ______
Do you believe you have a problem with morphine? ______
Any other comments that you would like to share about your use of morphine? (optional) ______

Fentanyl

How do you source your fentanyl? ______
How often do you use fentanyl? ______
How would you describe your use of fentanyl? ______
Fentanyl makes my eating disorder symptoms better. ______
Fentanyl makes my eating disorder symptoms worse. ______
Fentanyl has overall benefits for my mental health. ______
Fentanyl has unpleasant side effects. ______
Do you believe you have a problem with fentanyl? ______

Any other comments that you would like to share about your use of fentanyl? (optional) ______

Methadone

How do you source methadone? ______
How often do you use methadone? ______
Please describe your use of methadone. ______
Methadone makes my eating disorder symptoms better. ______
Methadone makes my eating disorder symptoms worse. ______
Methadone has overall benefits for my mental health. ______
Methadone has unpleasant side effects. ______
Do you believe you have a problem with methadone? ______
Any other comments that you would like to share about your use of methadone? (optional) ______

Oxycodone

How do you source oxycodone? ______
How often do you use oxycodone? ______
Please describe your use of oxycodone. ______
Oxycodone makes my eating disorder symptoms better. ______
Oxycodone makes my eating disorder symptoms worse. ______
Oxycodone has overall benefits for my mental health. ______
Oxycodone had unpleasant side effects. ______
Do you believe you have a problem with oxycodone? ______
Any other comments that you would like to share about your use of oxycodone? (optional) ______

Hydrocodone

How do you source hydrocodone? ______
How often do you use hydrocodone? ______
Please describe your use of hydrocodone. ______
Hydrocodone makes my eating disorder symptoms better. ______
Hydrocodone makes my eating disorder symptoms worse. ______
Hydrocodone has overall benefits for my mental health. ______
Hydrocodone has unpleasant side effects. ______
Do you believe you have a problem with hydrocodone? ______
Any other comments that you would like to share about your use of hydrocodone? (optional) ______

Hydromorphone

How do you source hydromorphone? ______
How often do you use hydromorphone? ______
Please describe your use of hydromorphone. ______
Hydromorphone makes my eating disorder symptoms better. ______
Hydromorphone makes my eating disorder symptoms worse. ______
Hydromorphone has overall benefits for my mental health. ______
Hydromorphone has unpleasant side effects. ______
Do you believe you have a problem with hydromorphone? ______
Any other comments that you would like to share about your use of hydromorphone? (optional) ______

Tapentadol

How do you source Tapentadol? ______
How often do you use Tapentadol? ______
Please describe your use of Tapentadol. ______
Tapentadol makes my eating disorder symptoms better. ______
Tapentadol makes my eating disorder symptoms worse. ______
Tapentadol has overall benefits for my mental health. ______
Tapentadol has unpleasant side effects. ______
Do you believe you have a problem with Tapentadol? ______
Any other comments that you would like to share about your use of Tapentadol? (optional) ______

Codeine

How do you source codeine? (select all that apply) ______
How often do you use codeine? ______
Please describe your use of codeine. ______
Codeine makes my eating disorder symptoms better. ______
Codeine makes my eating disorder symptoms worse. ______
Codeine has overall benefits for my mental health. ______
Codeine has unpleasant side effects. ______
Do you believe you have a problem with codeine? ______
Any other comments that you would like to share about your use of codeine? (optional) ______

Tramadol

How do you source tramadol? ______
How often do you use tramadol? ______
Please describe your use of tramadol. ______
Tramadol makes my eating disorder symptoms better. ______
Tramadol makes my eating disorder symptoms worse. ______
Tramadol has overall benefits for my mental health. ______
Tramadol has unpleasant side effects. ______
Do you believe you have a problem with tramadol? ______
Any other comments that you would like to share about your use of tramadol? (optional) ______

Other opioid

Name of opioid. ______
How do you source this opioid? ______
How often do you use this opioid? ______
Please describe your use of this opioid. ______
This opioid makes my eating disorder symptoms better. ______
This opioid makes my eating disorder symptoms worse. ______
This opioid has overall benefits for my mental health. ______
This opioid has unpleasant side effects. ______
Do you believe you have a problem with this opioid? ______
Any other comments that you would like to share about your use of this opioid ? (optional) ______

NOTE: YOU CAN EDIT YOUR PREVIOUS RESPONSES BY CLICKING ON THE SURVEY QUEUE ICON ON THE TOP RIGHT CORNER OF THIS PAGE.

Section 12: Other drugs

92% Complete

SECTION 12: OTHER DRUGS

The following questions will ask about other drugs that you may have used in the past 12 months that have NOT already been asked in this survey.

These may include but are not limited to:

Kratom, Benzodiazepines that you have sourced illegally (e.g xanax, roofies, valium) Khat, Xenon gas, Inhalable solvents (e.g. glue, paint, petrol), BZP (e.g. piperazines), Steroids and other performance enhancing drugs, Synthetic drugs sourced over the internet etc. Please note that this does not include synthetic cannabis. If you have used synthetic cannabis please ensure you have mentioned this in Section 7 Cannabis.

Are there any other drugs that you have used in the past 12 months that we have not included in this survey?	◯ Yes<br>◯ No
How many different other drugs have you used?	◯ 1<br>◯ 2<br>◯ 3<br>◯ 4<br>◯ 5+

Drug 1

Please name the drug. ______
Please describe your use of this drug. ______
How often do you use this drug? ______
This drug makes my eating disorder symptoms better. ______
This drug makes my eating disorder symptoms worse. ______
This drug has overall benefits for my mental health. ______
This drug has unpleasant side effects. ______
Do you believe you have a problem with this drug? ______
Any other comments that you would like to share about your use of this drug? (optional) ______

Drug 1

Please name the drug. ______
Please describe your use of this drug. ______
How often do you use this drug? ______
This drug makes my eating disorder symptoms better. ______
This drug makes my eating disorder symptoms worse. ______
This drug has overall benefits for my mental health. ______
This drug has unpleasant side effects. ______
Do you believe you have a problem with this drug? ______
Any other comments that you would like to share about your use of this drug? (optional) ______

Drug 2

Please name the drug. ______
Please describe your use of this drug. ______
How often do you use this drug? ______
This drug makes my eating disorder symptoms better. ______
This drug makes my eating disorder symptoms worse. ______
This drug has overall benefits for my mental health. ______
This drug has unpleasant side effects. ______

Do you believe you have a problem with this drug? ______
Any other comments that you would like to share about your use of this drug? (optional) ______

Drug 1

Please name the drug. ______
Please describe your use of this drug. ______
How often do you use this drug? ______
This drug makes my eating disorder symptoms better. ______
This drug makes my eating disorder symptoms worse. ______
This drug has overall benefits for my mental health. ______
This drug has unpleasant side effects. ______
Do you believe you have a problem with this drug? ______
Any other comments that you would like to share about your use of this drug? (optional) ______
Drug 2

Please name the drug. ______
Please describe your use of this drug. ______
How often do you use this drug? ______
This drug makes my eating disorder symptoms better. ______
This drug makes my eating disorder symptoms worse. ______
This drug has overall benefits for my mental health. ______
This drug has unpleasant side effects. ______

Do you believe you have a problem with this drug? ______
Any other comments that you would like to share about your use of this drug? (optional) ______
Drug 3

Please name the drug. ______
Please describe your use of this drug. ______
How often do you use this drug? ______
This drug makes my eating disorder symptoms better. ______
This drug makes my eating disorder symptoms worse. ______
This drug has overall benefits for my mental health. ______
This drug has unpleasant side effects. ______
Do you believe you have a problem with this drug? ______
Any other comments that you would like to share about your use of this drug? (optional) ______

Drug 1

Please name the drug. ______
Please describe your use of this drug. ______
How often do you use this drug? ______
This drug makes my eating disorder symptoms better. ______
This drug makes my eating disorder symptoms worse. ______
This drug has overall benefits for my mental health. ______
This drug has unpleasant side effects. ______
Do you believe you have a problem with this drug? ______
Any other comments that you would like to share about your use of this drug? (optional) ______
Drug 2

Please name the drug. ______
Please describe your use of this drug. ______
How often do you use this drug? ______
This drug makes my eating disorder symptoms better. ______
This drug makes my eating disorder symptoms worse. ______
This drug has overall benefits for my mental health. ______
This drug has unpleasant side effects. ______

Do you believe you have a problem with this drug? ______
Any other comments that you would like to share about your use of this drug? (optional) ______
Drug 3

Please name the drug. ______
Please describe your use of this drug. ______
How often do you use this drug? ______
This drug makes my eating disorder symptoms better. ______
This drug makes my eating disorder symptoms worse. ______
This drug has overall benefits for my mental health. ______

This drug has unpleasant side effects. ______
Do you believe you have a problem with this drug? ______
Any other comments that you would like to share about your use of this drug? (optional) ______
Drug 4

Please name the drug. ______
Please describe your use of this drug. ______
How often do you use this drug? ______
This drug makes my eating disorder symptoms better. ______
This drug makes my eating disorder symptoms worse. ______
This drug has overall benefits for my mental health. ______
This drug has unpleasant side effects. ______
Do you believe you have a problem with this drug? ______
Any other comments that you would like to share about your use of this drug? (optional) ______

Drug 1

Please name the drug. ______
Please describe your use of this drug. ______
How often are you using this drug? ______
This drug makes my eating disorder symptoms better. ______
This drug makes my eating disorder symptoms worse. ______
This drug has overall benefits for my mental health. ______
This drug has unpleasant side effects. ______
Do you believe you have a problem with this drug? ______
Any other comments that you would like to share about your use of this drug? (optional) ______
Drug 2

Please name the drug. ______
Please describe your use of this drug. ______
How often are you using this drug? ______
This drug makes my eating disorder symptoms better. ______
This drug makes my eating disorder symptoms worse. ______
This drug has overall benefits for my mental health. ______
This drug has unpleasant side effects. ______

Do you believe you have a problem with this drug? ______
Any other comments that you would like to share about your use of this drug? (optional) ______
Drug 3

Please name the drug. ______
Please describe your use of this drug. ______
How often are you using this drug? ______
This drug makes my eating disorder symptoms better. ______
This drug makes my eating disorder symptoms worse. ______
This drug has overall benefits for my mental health. ______
This drug has unpleasant side effects. ______
Do you believe you have a problem with this drug? ______
Any other comments that you would like to share about your use of this drug? (optional) ______
Drug 4

Please name the drug. ______
Please describe your use of this drug. ______
How often are you using this drug? ______
This drug makes my eating disorder symptoms better. ______
This drug makes my eating disorder symptoms worse. ______
This drug has overall benefits for my mental health. ______
This drug has unpleasant side effects. ______
Do you believe you have a problem with this drug? ______
Any other comments that you would like to share about your use of this drug? (optional) ______
Drug 5

Please name the drug. ______
Please describe your use of this drug. ______
How often are you using this drug? ______
This drug makes my eating disorder symptoms better. ______
This drug makes my eating disorder symptoms worse. ______
This drug has overall benefits for my mental health. ______
This drug has unpleasant side effects. ______

Do you believe you have a problem with this drug? ______
Any other comments that you would like to share about your use of this drug? (optional) ______

Thank you for completing the past 11 sections on your use of prescription medication and/or drug use.

Do you have a prescription medication or drug of choice for treating your eating disorder? If yes can you please name your top three in order of preference (1st being the most preferred).

1st ______
2nd ______
3rd ______
(optional)

Irrespective of your eating disorder what is your drug of choice? Can you please name your top three in order of preference (1st being the most preferred).

1st ______
2nd ______
3rd ______
(optional)

NOTE: YOU CAN EDIT YOUR PREVIOUS RESPONSES BY CLICKING ON THE SURVEY QUEUE ICON ON THE TOP RIGHT CORNER OF THIS PAGE.

End

100% Complete

Thank you for your contribution to this research.

We recognise this survey may have raised difficult issues for you.

Please consider reaching out to supports such as:

a GP, counsellor, psychologist or psychiatrist family or friends school or college counsellor a teacher or coach a work colleague

---

Australian Support Services:

If you need someone to talk to, please consider contacting one of the following free 24-hour support services available in Australia:

• Lifeline 24-hour Australian crisis counselling (Telephone: 13 11 13; website: https://www.lifeline.org.au/)
• Beyond Blue 24-hour phone support (Telephone 1300 22 4636; https://www.beyondblue.org.au/)

It may help to speak with a general practitioner about creating a mental health plan: https://www.servicesaustralia.gov.au/individuals/subjects/whats-covered-medicare/mental-health-care-and-medicare

The Australian Psychological Society 'Find a Psychologist' service can connect you with a psychologist by issue and location: https://psychology.org.au/find-a-psychologist

The Black Dog Institute website also has a wide range of resources to help keep you safe: https://www.blackdoginstitute.org.au/resources-support/suicide-self-harm/support-services/

Alcohol and Drug Support Services for each state:

WA Alcohol and Drug Support (ADIS) 24-hour support line T: 1800 198 024 (regional) or (08) 9442 5000 (Metropolitan) NSW Alcohol and Drug Support (ADIS) 24-hour support line T: 1800 250 015 or 1800 422 599 (regional) or (02) 9361 8000 (Metropolitan), webchat: www.yourroom.health.nsw.gov.au/webchat QLD Alcohol and Drug Support (ADIS) 24-hour support line T: 1800 177 833 (regional) or (07) 3837 5989 (Metropolitan), website: https://adis.health.qld.gov.au/ SA Alcohol and Drug Support (ADIS) 8:30am - 10:00pm T: 1300 131 340 (local call fee). Interstate callers can contact ADIS on (08) 7087 1743 ACT Alochol and Drug Support ADIS T: (02) 6207 9977 Alcohol Tobacco & Other Drug Association ACT 24/7 Phone Line T: (02) 5124 9977 (ask for the Alcohol and Drug Services) NT Alcohol and Drug Support (ADIS) 24/7 Phone line T:1800 131 350 Drug and Alcohol Clinical Advisory Service (DACAS) T: 1800 111 092, website: https://www.dacas.org.au/ Tasmania Alcohol and Drug Support (ADIS) 24/7 Phone line T: 1800 811 994 VIC DirectLine 24/7 Phone line T: 1800 888 236, website: https://www.directline.org.au/ Drug and alcohol clinical advisory service (DACAS) 24 hour support line T:1800 812 804, website: https://www.dacas.org.au/ National alcohol and drug online counselling service https://www.counsellingonline.org.au/

---

for SurveyCircle users (www.surveycircle.com): The Survey Code is: ENS3-N8S4-KG43-J73C
Redeem Survey Code with one click: https://www.surveycircle.com/ENS3-N8S4-KG43-J73C

for SurveySwap users go to: https://surveyswap.io/sr/65KF-DRIC-VLBU

Or, alternatively, enter the code manually: 65KF-DRIC-VLBU

eTable 1. Retention of Respondents Through the Sections of the Survey by Diagnosis

**Survey Section**	**All**	**UD**	**AN**	**AN+BN**	**BN**	**BED**	**ARFID**	**OSFED**
Consent Page	7648							
1. Demographics	6612	2493	1485	425	282	261	158	214
2. ED Details, Diagnosis and Treatment	6612	2493	1485	425	282	261	158	214
3. Prescription Medications	6136	2358	1376	388	260	242	139	194
4. Caffeine	5864	2244	1323	367	247	230	134	187
5. Alcohol	5722	2189	1290	355	239	223	132	183
6. Tobacco + E-cigarettes	5606	2129	1277	347	233	218	128	181
7. Cannabis	5383	2032	1234	327	224	211	122	178
8. Stimulants	5276	1988	1209	319	217	208	120	176
9. Psychedelics	5247	1977	1202	317	216	205	120	176
10. Pro-social/Party Drugs (MDMA, ketamine, GHB)	5201	1960	1189	315	212	205	117	175
11. Opioids	5184	1950	1188	314	212	205	117	172
12. Other drugs	5123	1923	1173	311	210	203	116	172

eTable 1. Retention numbers through various sections of survey for all respondents and per diagnosis (N>150)
Abbreviations: ALL = All respondents, UN = Undiagnosed, AN = Anorexia Nervosa, BN = Bulimia Nervosa, BED = Binge Eating Disorder, ARFID = Avoidant/Restrictive Feeding Intake Disorder, OSFED = Other Specified Feeding or Eating Disorder, UFED = Unspecified Feeding or Eating Intake Disorder, RD = Rumination Disorder, ED = Eating Disorder, MDMA = 3,4-methylenedioxymethamphetamine, GHB = Gamma-hydroxybutyrate

© 2025 Rodan SC et al. *JAMA Network Open*.

eTable 2. Frequency of Distinct Diagnostic Categories

	Diagnosis	Frequency	%
1	Undiagnosed/ Disordered eating	2493	37.70
2	AN	1485	22.46
3	AN BN	425	6.43
4	BN	282	4.26
5	BED	261	3.95
6	OSFED	214	3.24
7	ARFID	158	2.39
8	AN ARFID	148	2.24
9	BN BED	141	2.13
10	AN OSFED	138	2.09
11	UFED	118	1.78
12	AN BN BED	97	1.47
13	AN BED	82	1.24
14	AN BN OSFED	57	0.86
15	AN BN ARFID	48	0.73
16	BN OSFED	40	0.60
17	BED ARFID	33	0.50
18	AN UFED	28	0.42
19	AN BED ARFID	23	0.35
20	BN ARFID	23	0.35
21	OSFED ARFID	23	0.35
22	UFED ARFID	22	0.33
23	AN BN BED ARFID	18	0.27
24	BN BED ARFID	14	0.21
25	AN BN BED OSFED	12	0.18
26	AN BN OSFED ARFID	12	0.18
27	AN BN UFED	12	0.18
28	AN OSFED ARFID	12	0.18
29	AN RD	11	0.17
30	BED OSFED	11	0.17
31	BED UFED	11	0.17
32	AN BED OSFED	10	0.15
33	AN BN RD	9	0.14
34	AN UFED ARFID	9	0.14
35	BN UFED	9	0.14
36	OSFED UFED	9	0.14
37	AN PICA	8	0.12
38	BN BED UFED	7	0.11
39	AN BN PICA	6	0.09
40	AN ARFID PICA	5	0.08
41	BN BED OSFED	5	0.08
42	AN ARFID RD	4	0.06
43	AN BN BED OSFED ARFID	4	0.06
44	ARFID RD	4	0.06
45	BN RD	4	0.06
46	AN BN OSFED UFED	3	0.05

© 2025 Rodan SC et al. *JAMA Network Open*.

47	AN OSFED UFED	3	0.05
48	RD	3	0.05
49	AN BED UFED	2	0.03
50	AN BN ARFID PICA	2	0.03
51	AN BN ARFID RD	2	0.03
52	AN BN BED RD	2	0.03
53	AN BN BED UFED	2	0.03
54	AN BN BED UFED ARFID	2	0.03
55	AN BN OSFED UFED ARFID	2	0.03
56	ARFID PICA	2	0.03
57	BED OSFED UFED	2	0.03
58	BED PICA	2	0.03
59	BN BED OSFED ARFID	2	0.03
60	BN PICA	2	0.03
61	OSFED ARFID PICA	2	0.03
62	OSFED PICA	2	0.03
63	OSFED UFED ARFID	2	0.03
64	UFED PICA	2	0.03
65	AN BED OSFED ARFID	1	0.02
66	AN BED OSFED UFED ARFID PICA	1	0.02
67	AN BED PICA	1	0.02
68	AN BN BED OSFED RD	1	0.02
69	AN BN BED OSFED UFED	1	0.02
70	AN BN BED OSFED UFED ARFID	1	0.02
71	AN BN OSFED UFED PICA	1	0.02
72	AN OSFED ARFID PICA	1	0.02
73	AN OSFED ARFID RD	1	0.02
74	AN OSFED PICA	1	0.02
75	AN OSFED RD	1	0.02
76	AN OSFED UFED ARFID	1	0.02
77	ARFID RD PICA	1	0.02
78	BED OSFED ARFID	1	0.02
79	BED UFED ARFID	1	0.02
80	BED UFED PICA	1	0.02
81	BN ARFID PICA	1	0.02
82	BN BED ARFID PICA	1	0.02
83	BN BED OSFED UFED	1	0.02
84	BN BED PICA	1	0.02
85	BN BED RD	1	0.02
86	BN OSFED RD	1	0.02
87	BN UFED ARFID	1	0.02
88	BN UFED PICA	1	0.02
89	BN UFED RD	1	0.02
90	OSFED RD	1	0.02

eTable 2. Numbers of respondents self-reporting each type of diagnoses or co-diagnoses reported in the survey. The undiagnosed category is also included. Abbreviations: AN = Anorexia Nervosa, BN = Bulimia Nervosa, BED = Binge Eating Disorder, ARFID = Avoidant/Restrictive Feeding Intake Disorder, OSFED = Other

Specified Feeding or Eating Disorder, UFED = Unspecified Feeding or Eating Intake Disorder, RD = Rumination Disorder

© 2025 Rodan SC et al. *JAMA Network Open*.

eTable 3. Top Drugs of Choice Nominated by Respondents

	N nominating as 1st choice	N (%) completing question	% of completers naming drug as first choice
**Drug of choice for treating ED[a]**			
1. Cannabis	452	918/3018 (30.4)	49.2
2. Fluoxetine	119	200/582 (34.4)	60.0
3. Sertraline	82	209/678 (30.8)	39.2
4. Lisdexamfetamine	75	139/209 (66.5)	54.0
5. Psilocybin	40	309/807 (38.3)	12.9
**Overall drug of choice[b]**			
1. Cannabis	852	2149/3018 (71.2)	39.6
2. Caffeine	129	2092/5676 (36.9)	6.2
3. Alcohol	75	1885/4759 (39.6)	4.0
4. Nicotine	84	1202/2669 (45.0)	7.0
5. Psilocybin	61	520/807 (64.4)	11.7

eTable 3. Respondents were given the option to rate their top 3 “drugs of choice” for treating their ED and their overall “drug of choice” irrespective of their ED. The relevant questions were:

[a] Do you have a prescription medication or drug of choice for treating your eating disorder? If yes can you please name your top three in order of preference (1st being the most preferred)

[b] Irrespective of your eating disorder what is your drug of choice? Can you please name your top three in order of preference (1st being the most preferred)

The Table shows the top five drugs by number of respondents naming that drug as their drug of choice, as well as the number of total users of that drug completing the question, and the percentage completing the question who nominated that drug as their first choice.

Abbreviations: ED = Eating Disorder

© 2025 Rodan SC et al. *JAMA Network Open*.

eTable 4. Most Problematic Drugs Nominated by Respondents

	Total N	N (%) reporting a Problem	N (%) of reporting problematic use using daily	Total N (%) reporting daily use
Nicotine	2669	1381 (51.7)	1304 (94.4)	1762 (66.0)
Caffeine	5676	955 (16.8)	904 (94.7)	4388 (77.3)
Tobacco	2359	840 (35.6)	607 (72.3)	881 (37.3)
Alcohol	3512	636 (18.1)	126 (19.8)	178 (5.1)
Cannabis	3018	556 (18.4)	455 (81.8)	1272 (42.2)
Cocaine	787	68 (8.6)	10 (6.8)	13 (1.7)
Codeine	746	48 (6.4)	17 (35.4)	65 (8.7)
Ketamine	573	46 (8.0)	18 (39.1)	20 (3.5)
MDMA	839	44 (5.2)	1 (2.3)	1 (0.1)
Amphetamine	280	36 (12.9)	6 (16.7)	10 (3.6)

eTable 4. Top 10 drugs with highest frequency of respondents self-reporting a problem with that drug. For each drug used, respondents were asked "do you believe you have a problem with X"

Abbreviation: MDMA = 3,4-methylenedioxymethamphetamine

© 2025 Rodan SC et al. *JAMA Network Open*.
