# Supplementary material for: Prescription and Nonprescription Drug Use Among People With Eating Disorders
Source: JAMA Netw Open. 2025 Jul 22;8(7):e2522406. doi: 10.1001/jamanetworkopen.2025.22406 (PMC12284744; doi:10.1001/jamanetworkopen.2025.22406)
Supplement: Supplement 2. — Data Sharing Statement [file jamanetwopen-e2522406-s002.pdf]

Data Sharing Statement

Rodan. Prescription and Nonprescription Drug Use Among People With Eating Disorders. *JAMA Netw Open*. Published July 22, 2025. doi:10.1001/jamanetworkopen.2025.22406

Data
Data available: No

Additional Information
Explanation for why data not available: Reasonable requests for data will be considered
